# FtsK with a unique N-terminal extension is involved in coordinating the final steps of chromosome segregation with asymmetric division in mycobacterial cells

**DOI:** 10.1128/jb.00096-26

**Published:** 2026-05-29

**Authors:** Kornel Milcarz, Joanna Hołówka, Damian Trojanowski, Tomasz Łebkowski, Dominik Bania, Michał Tracz, Jolanta Zakrzewska-Czerwińska

**Affiliations:** 1 Department of Molecular Microbiology, University of Wrocław 49572, Wrocław, Poland; 2 Protein Mass Spectrometry Laboratory, University of Wrocław 49572, Wrocław, Poland; University of Notre Dame, Notre Dame, Indiana, USA

**Keywords:** *Mycobacterium smegmatis*, DNA translocation, cell division, cell cycle, FtsK, fluorescence microscopy

## Abstract

In this study, we identify and functionally characterize a previously unrecognized, conserved N-terminal extension of mycobacterial FtsK (nFtsK) that contributes to the coordination of chromosome segregation with asymmetric cell division. Although FtsK is broadly conserved in bacteria as a late-stage DNA translocase and divisome component, mycobacterial FtsK uniquely contains a long, positively charged, intrinsically disordered N-terminal region. We show that this extension is dispensable for core FtsK functions—including septal localization, DNA translocation, and completion of cell division—but instead modulates the spatial confinement, temporal regulation, and interaction specificity of these processes. Mechanistically, nFtsK mediates specific protein interactions, binds the anionic phospholipids cardiolipin and phosphatidic acid, and exhibits intrinsic affinity for negatively curved membranes. Together, our findings uncover a lineage-specific adaptation that fine-tunes a conserved molecular machine, enhancing the robustness of asymmetric cell division in mycobacteria.

**IMPORTANCE:**

Faithful cell division requires precise coordination between chromosome segregation and septum formation. Mycobacteria lack the canonical spatial regulators found in many bacteria, raising the question of how this coordination is achieved in asymmetrically dividing cells. Here, we identify a lineage-specific N-terminal extension of the DNA translocase FtsK that contributes to spatial control during mycobacterial division. This intrinsically disordered region associates with anionic lipids and curved membrane regions at the septum, coupling chromosome translocation to septal maturation. Our findings reveal how mycobacteria adapt conserved DNA translocation machinery to their unconventional division mode.

## INTRODUCTION

Most rod-shaped bacteria grow by lateral elongation and divide symmetrically via septum formation at mid-cell, producing two daughter cells of equal length, each inheriting a complete copy of the genome. These processes have been extensively studied in model organisms, such as the Gram-negative *Escherichia coli* and the Gram-positive *Bacillus subtilis*. In these species, symmetric division is tightly regulated by spatial control systems—including the MinCDE and nucleoid occlusion (NO) systems—that ensure accurate placement of the FtsZ ring (1, 2). These mechanisms prevent mispositioning of the division machinery and protect against lethal guillotining of the chromosome(s). In contrast, *Mycobacterium* species exhibit a markedly different mode of growth and division. These bacteria elongate in a polar, biphasic manner, with the old pole growing more rapidly than the newly formed pole, and divide asymmetrically, generating daughter cells of unequal lengths and different physiology (3, 4). Notably, no homologs of the MinCDE or NO systems have been identified in mycobacteria to date, raising fundamental questions about how chromosome segregation and cell division are coordinated to preserve genome integrity.

The early steps of chromosome segregation in mycobacteria are relatively well characterized. The ParAB*S* system plays a central role in positioning newly replicated *oriC* regions toward the cell poles: ParB binds centromere-like *parS* sequences near *oriC* to form nucleoprotein complexes (segrosomes), which are actively segregated by the ATPase ParA (5, 6). However, the later steps of segregation—particularly DNA translocation across the closing septum—remain poorly understood.

In most bacteria, late-stage chromosome segregation is mediated by FtsK, an essential DNA translocase and core divisome component (7, 8). In *E. coli*, FtsK localizes to the septum during late cytokinesis, and deletion of its C-terminal motor domain results in defects in both chromosome segregation and septation. FtsK assembles into a hexameric, ATP-driven motor that actively translocates double-stranded DNA from the division site into the nascent daughter cell (9). Its C-terminal domain comprises three subdomains: α and β, which form the hexameric motor, and γ, which controls directionality and interacts with the XerCD recombinase complex (10). FtsK recognizes G-rich KOPS (FtsK orienting polar sequences) motifs that guide DNA translocation toward the *dif* site near the replication terminus (*ter*) region (11, 12). These motifs are asymmetrically distributed along each chromosome arm, oriented toward *dif* and progressively enriched. Translocation terminates through interaction with the XerCD recombinase complex, facilitating chromosome dimer resolution and decatenation (10). Beyond translocase activity, the N-terminal domain of FtsK anchors the protein to the cytoplasmic membrane via transmembrane helices and mediates interactions with other divisome components (e.g., FtsQ, FtsL, FtsI), thereby coupling cell division with chromosome segregation (13). Studies in other organisms (e.g., *Staphylococcus aureus*, *Deinococcus radiodurans*) further demonstrate that FtsK indirectly influences the levels of the septal peptidoglycan hydrolase Sle1, divisome assembly, and the timing of septal splitting, underscoring its dual role in DNA translocation and coordination of cell division (14).

**Fig 1:**
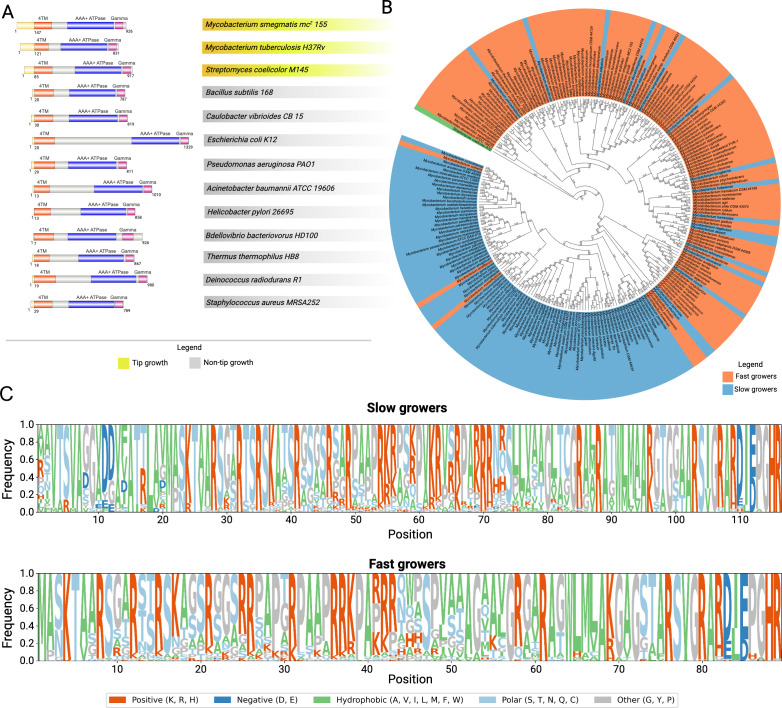
Comparative analysis and conservation of FtsK across diverse bacterial species and within the *Mycobacterium* genus. (**A**) Comparison of FtsK domain organization in *Mycobacterium* species and representative bacterial model organisms. The N-terminal extension is indicated in yellow, followed by four transmembrane helices (4TM) shown in orange. The linker region is depicted in gray, while the AAA+ ATPase motor domain and the gamma domain, responsible for KOPS interaction, are shown in blue and violet, respectively. (**B**) Phylogenetic tree based on an alignment of FtsK sequences from *Mycobacteriaceae* species, with color-coded grouping according to growth rate (fast—orange; slow—blue). *Streptomyces coelicolor* A3(2) (green) was used as an outgroup for rooting. (**C**) Sequence logo of the nFtsK consensus for slow- and fast-growing *Mycobacteriaceae* species, showing enrichment of positively charged and hydrophobic residues.

In mycobacteria, FtsK is essential (15–18), but it remains largely uncharacterized. Notably, mycobacterial FtsK contains a uniquely extended, intrinsically disordered, and positively charged N-terminal region (see Fig. 1A), suggesting a lineage-specific adaptation linked to polar growth and asymmetric division. Here, we show that this conserved N-terminal region enhances the robustness and precision of late chromosome segregation by constraining translocase dynamics and septal positioning. Loss of this region does not abolish FtsK function but leads to faster, yet less precisely coordinated, cell cycle progression.

## RESULTS

### *In silico* analysis reveals a conserved N-terminal extension in mycobacterial FtsK

Variation among FtsK proteins is primarily observed in their N-terminal domains, which typically contain four transmembrane helices and a linker connecting the membrane anchor to the well-conserved cytosolic translocation motor (19). To assess whether mycobacterial FtsK differs from homologs in other bacteria, we compared amino acid sequences from a few representative bacterial species. Sequence alignment revealed that mycobacterial FtsK possesses a unique, extended N-terminal tail (Fig. 1A and B). In most Gram-positive and Gram-negative bacteria, the first transmembrane helix begins immediately after the initiator methionine. In contrast, mycobacterial FtsK contains an additional N-terminal extension of up to 150 amino acids upstream of these helices (Fig. 1A). A similar but shorter extension (approximately 80 amino acids) was observed in *Streptomyces coelicolor*, another member of the phylum Actinomycetota.

This N-terminal extension (hereafter referred to as nFtsK) is conserved across mycobacteria, with modest variation in length. Analysis of 226 full-length FtsK sequences from Mycobacteriaceae revealed that the nFtsK extension is longer in slow-growing pathogenic species compared to fast-growing species (Fig. 1B). The length of nFtsK differs significantly between these groups, with slow growers encoding longer extensions than fast growers (Fig. S1A; *P* = 1.49 × 10⁻⁴). *Mycobacterium smegmatis* is a notable exception: its nFtsK (~121 aa) equals or exceeds those of many slow-growing species, including *M. tuberculosis* (~95 aa), suggesting a lineage-specific expansion within this clade (Fig. 1A and Fig. S1A).

In *M. smegmatis*, the nFtsK extension is strongly positively charged (net charge +23, pI = 12) and hydrophilic, enriched in Arg (15%), Lys (3.9%), Ser (10%), and Gly (10%), suggesting that this segment is intrinsically disordered. Sequence logo analysis of the nFtsK consensus across all 226 sequences confirmed that the enrichment of positively charged residues is a broadly conserved feature of this extension, with Arg and Lys residues occurring at high frequency in both slow- and fast-growing species (Fig. 1C). The nFtsK extension is present in saprophytic and pathogenic mycobacteria, indicating that it is not restricted to a particular lifestyle and likely represents an ancestral feature of the genus.

In summary, mycobacterial FtsK harbors a conserved, positively charged, and hydrophilic N-terminal extension that is absent from most bacterial lineages. Although nFtsK is, on average, longer in slow-growing species, *M. smegmatis* stands out among fast-growing species by harboring an unusually elongated nFtsK, pointing to a lineage-specific structural elaboration in this saprophyte.

### Truncation of the N-terminal extensions alters FtsK dynamics and accelerates the cell cycle

Because nFtsK is conserved among mycobacteria regardless of their lifestyle (Fig. 1), we next investigated its role using *M. smegmatis mc² 155* as a model organism. We initially attempted to delete the 5′-end fragment of the *ftsK* gene encoding the entire unstructured N-terminal region. However, no viable double-crossover (DCO) strains were obtained, suggesting that the transmembrane α-helix extends further toward the N-terminus than predicted by secondary structure analysis. We therefore constructed a truncated variant lacking only the first 140 amino acids, retaining additional residues to ensure the integrity of the first predicted transmembrane α-helix. This variant, termed sFtsK (short FtsK), was also fused to a HaloTag (sFtsK-HT) and compared with either WT or FtsK-HT, respectively.

**Fig 2:**
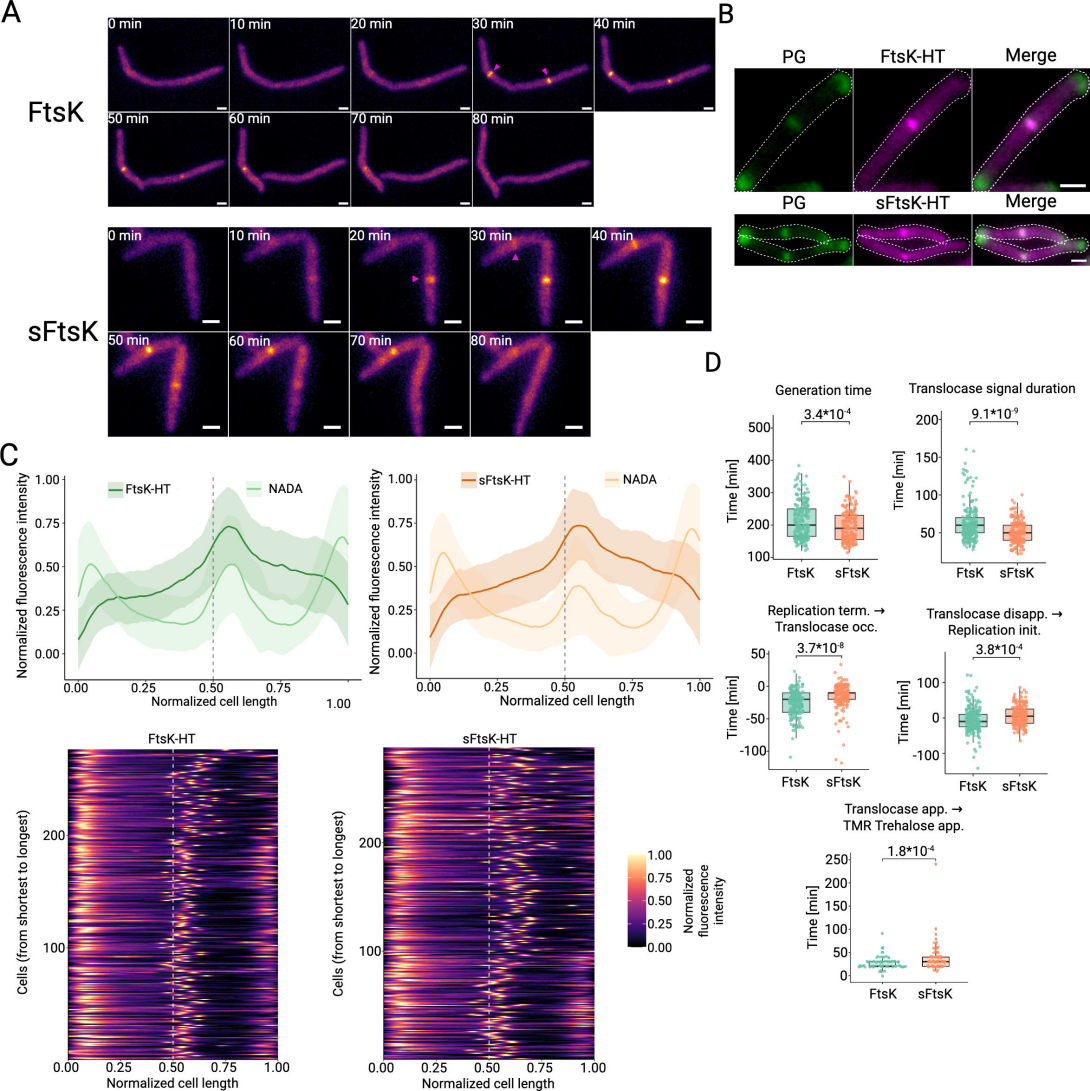
The truncated sFtsK variant exhibits altered localization and accelerated cell cycle dynamics. (**A**) Representative time-lapse fluorescence microscopy images of *M. smegmatis* cells expressing FtsK-HT (top panels) or the truncated sFtsK-HT variant (bottom panels). Magenta arrowheads indicate the appearance of representative translocase foci. Scale bar, 1 µm. (**B)** Representative images showing colocalization of sFtsK-HT with the peptidoglycan marker NADA-Green; scale bar, 1 µm. (**C)** Population-level profiles of normalized sFtsK-HT or FtsK-HT and NADA (cell wall marker) fluorescence intensity plotted against normalized cell length (upper panel), showing that loss of nFtsK results in a slightly broader fluorescence intensity peak shifted closer to mid-cell (0.50 of normalized cell length) (*n* = 87 and 91 for FtsK-HT and sFtsK-HT strains, respectively). The lower panel shows a heatmap of normalized fluorescence intensities of NADA-stained cells plotted against normalized cell length. Cells on the y-axis are sorted from shortest to longest. The sFtsK-HT strain shows a less focused septal signal, with a wider distribution that increases with cell length. (**D)** Quantification of cell cycle parameters and translocase dynamics in FtsK-HT (teal) and sFtsK-HT (orange) strains. Plots show generation time (top left), translocase signal duration (top right), the time interval between replication termination and translocase appearance (bottom left), the time interval between translocase disappearance and subsequent replication initiation (bottom right), and the time interval between translocase appearance and TMR trehalose appearance (cell envelope marker). Each dot represents a single cell.

The sFtsK-HT strain exhibited normal morphology but displayed a modest yet significant reduction in mean cell length compared with the FtsK-HT strain (3.75 ± 1.00 µm vs 4.21 ± 1.01 µm; *P* = 1.6 × 10^−3^, *n* = 104). Both strains exhibited comparable growth profiles; however, HaloTag fusions showed a reduction in growth rate relative to the wild-type and sFtsK strain (Fig. S1B). FtsK-HT and sFtsK-HT formed distinct signals at the septum (Fig. 2A) and colocalized with the septal marker (Fig. 2B), indicating that the N-terminal extension is not required for septal recruitment. Interestingly, the population-level analysis revealed that sFtsK-HT localized slightly closer to midcell compared with the full-length protein (Fig. 2C, upper panel), suggesting reduced spatial confinement in the absence of the N-terminal extension of FtsK. Further analysis showed that the septum (NADA-stained) and translocase signals did not differ in their maximum peak positions (Fig. S2A). Heatmap analysis of individual cells revealed that FtsK-HT remained within a similar range across normalized cell lengths, a trend also observed without normalization (Fig. S2B). In contrast, the nFtsK truncation broadened the distribution of septum placement, and the longest cells exhibited increased variance in septum positioning (Fig. 2C, lower panel). Because septum positioning differed between strains, we investigated whether cells inheriting the pole from the previous division (new-pole cells) versus those inheriting the older pole (old-pole cells) exhibited differences. Both groups of mutant cells were significantly shorter than FtsK-HT cells (3.51 ± 0.60 µm vs 3.28 ± 0.60 µm, *P* = 6.40 × 10^−5^ for old pole cells; 3.05 ± 0.54 µm vs 2.91 ± 0.49 µm, *P* = 6.20 × 10^−4^ for new pole cells; *n* = 276 and 287 for FtsK-HT and sFtsK-HT strain, respectively) (Fig. S2C). Cell asymmetry, expressed as the old-pole/new-pole length ratio, showed a trend toward a broader distribution in the mutant (Fig. S2C). FtsK residence time at the septum was also reduced upon removal of the N-terminal extension. Full-length FtsK-HT persisted at the septum for 62 ± 24 min, whereas sFtsK-HT remained for only 50 ± 14 min (*P* = 2.23 × 10^−8^; *n* = 216 and 189, respectively) (Fig. 2D, top right). Importantly, cellular levels of full-length FtsK-HT and the truncated variant were comparable (Fig. S3).

To place these localization differences in the context of the cell cycle, we monitored DNA replication using the replisome marker DnaN-mCherry (20), which allowed tracking of the C phase (replication duration) and the BD phase (the interval between replication termination and initiation of the next round in daughter cells). Generation time, defined as the interval between replication focus appearance in mother and daughter cells, was significantly shorter in sFtsK-HT-expressing cells than in FtsK-HT cells (195 ± 47 min vs 214 ± 56 min; *P* = 3.96 × 10^−4^; *n* = 189 and 216, respectively) (Fig. 2D, top left), consistent with the reduced duration of sFtsK septal localization. Relative to replication termination, FtsK-HT foci appeared 26 ± 18 min before termination, whereas sFtsK-HT foci emerged only 15 ± 18 min earlier (*P* = 3.62 × 10⁻⁸, *n* = 216 and 189, for FtsK-HT and sFtsK-HT, respectively) (Fig. 2D, bottom left). In daughter cells, replication initiation occurred later in the sFtsK-HT strain, whereas in FtsK-HT cells it tended to begin slightly before translocase disappearance (6 ± 27 min vs −5 ± 34 min; *P* = 4.63 × 10⁻⁴, *n* = 189 and 216, for sFtsK-HT and FtsK-HT, respectively) (Fig. 2D, bottom right). Notably, both strains exhibited substantial heterogeneity, reflecting the flexible coupling between DNA replication and cytokinesis in *Mycobacterium*. We next assessed whether this flexibility extended to cell envelope biogenesis by measuring septal TMR-trehalose incorporation (Fig. 2D, bottom center). While both strains showed similar modes of signal appearance, incorporation was significantly delayed and more heterogeneous in sFtsK-HT cells compared with the full-length FtsK cells (38 ± 26 min vs 27 ± 12 min; *P* = 1.80 × 10^−4^, *n* = 110 and 107, respectively). The increased variance in sFtsK-HT cells is consistent with impaired temporal coordination between septal cell wall synthesis and the cell cycle following removal of the N-terminal extension.

Given these temporal shifts, we next tested whether the coordination between translocation termination and chromosome segregation is altered in sFtsK-HT cells. Using the nucleoid marker HupB (21) as a proxy for chromosome segregation, we measured the interval between translocase disassembly and HupB splitting in individual cells. The mean interval did not differ between variants (−0.1 ± 14.1 min for FtsK-HT vs −0.9 ± 16.8 min for sFtsK-HT; Welch’s *t*-test, *P* = 0.667), indicating that average timing is unaffected by removal of the N-terminal extension. However, the cell-to-cell variability of this interval was significantly increased in sFtsK-HT cells (variance: 281 vs 198; Levene’s test, *P* = 0.023; *n* = 120 and 134 for sFtsK-HT and FtsK-HT, respectively; Fig. S4A). Thus, the N-terminal extension does not set the average timing of chromosome segregation relative to cytokinesis but limits cell-to-cell variability. Consistently, nFtsK enhances the robustness of this coordination without affecting its mean timing. Importantly, live/dead staining analysis showed that removal of nFtsK did not increase the proportion of anucleated or dead cells (Fig. S4B).

Together, these data indicate that the nFtsK extension is dispensable for septal recruitment and overall divisome function. However, its removal shortens FtsK septal residence time, accelerates the cell cycle, and shifts the timing of FtsK assembly relative to DNA replication and septal cell wall synthesis. While the mean interval between translocation termination and chromosome segregation is preserved, cell-to-cell variability increases, indicating that nFtsK constrains stochastic fluctuations in the coupling of these processes.

### The N-terminal extension constrains FtsK diffusion and spatial confinement

**Fig 3:**
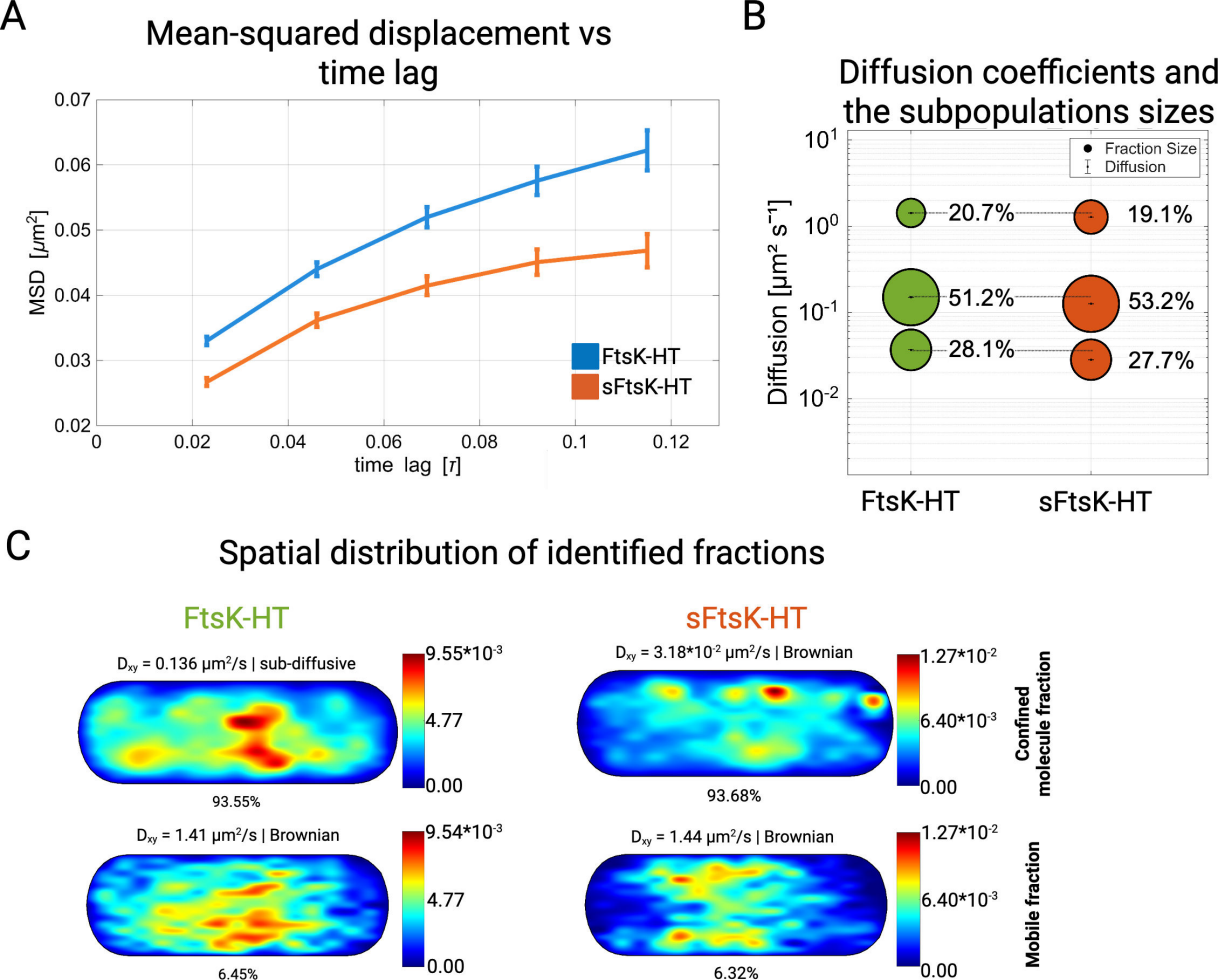
Single particle tracking analysis of FtsK-HT and sFtsK-HT. (**A)** Averaged mean squared displacement plotted as a function of time lag (τ). The sFtsK-HT protein (orange) exhibits a lower slope than FtsK-HT (blue), indicating reduced overall molecular mobility. Error bars represent standard deviation. (**B**) Bubble plot derived from squared displacement (SQD) analysis using non-simultaneous independent fitting for each strain, showing diffusion coefficients (D_xy_) and relative subpopulation sizes (bubble area). Horizontal lines indicate the diffusion coefficient of each subpopulation. Despite comparable subpopulation proportions, diffusion coefficients are consistently reduced across all three mobility states in sFtsK-HT (*P* = 8.67 × 10⁻⁶⁴, Kolmogorov-Smirnov test). (**C**) Spatial probability density heat maps from clustering analysis showing particle localization projected onto a normalized cell contour (blue = low, red = high density; color scales are independently normalized per condition). Upper panels (cluster 1, ~93.55% and 93.68% of tracks in FtsK-HT and sFtsK, respectively): the slow/confined population of FtsK-HT (D*_xy_* = 0.136 µm²/s; sub-diffusive) displays mid-cell accumulation consistent with septal confinement, whereas sFtsK-HT (D*_xy_* = 3.18 × 10^−2^ µm²/s; Brownian) shows a diffuse distribution lacking mid-cell enrichment. Lower panels (cluster 2, ~6.45% and 6.32 % of tracks in FtsK-HT and sFtsK-HT, respectively): mobile population displays Brownian motion in both variants; however, FtsK-HT trajectories retain a preferential enrichment in the mid-cell region.

To investigate the molecular basis of the altered cell cycle timing, we performed single-particle tracking (SPT) of FtsK-HT and the truncated variant sFtsK-HT. In total, 9,951 trajectories were analyzed for FtsK-HT and 8,843 for sFtsK-HT. These data sets were used in subsequent analyses. Mean squared displacement (MSD) analysis revealed similar overall diffusion trends for both variants: MSD increased with the time lag (τ), but its rate of increase decreased over time, consistent with subdiffusive behavior (Fig. 3A). Corresponding with the reduced MSD, the average diffusion coefficient was lower for sFtsK-HT than for FtsK-HT (D_xy_ = 8.90 × 10^−2^ µm^2^/s vs 6.60 × 10^−2^ µm^2^/s; tracks/cell = 59 and 76, *n* = 169 and 134, respectively) (Fig. 3A).

To further resolve diffusive heterogeneity, we performed squared displacement (SQD) analysis using a non-simultaneous parameter approach. Although the relative population sizes of diffusive states were similar between the two variants, their dynamic properties differed markedly (*P* = 8.67 × 10^−64^, Fig. 3B). Specifically, diffusion coefficients were consistently reduced for sFtsK-HT across all three mobility states. The fast mobile fraction slowed from D_xy_ = 1.430 ± 0.003 µm^2^/s in FtsK-HT to 1.280 ± 0.003 µm^2^/s in sFtsK-HT. Likewise, both the slow mobile (0.150 vs 0.126 µm^2^/s) and confined fractions (0.037 vs 0.028 µm^2^/s) exhibited reduced mobility in the absence of the extended N-terminal region. Despite these changes in diffusion rates, the relative proportions of the three populations remained comparable between variants (from most mobile to least mobile: 20.7% vs 19.1%, 51.2% vs 53.2%, 28.1% vs 27.7% for FtsK-HT and sFtsK-HT, respectively). The underlying single-step displacement distributions for both strains are shown in Fig. S5A.

We next assessed immobilization kinetics using dwell-time analysis with a 100-nm radius and a two-component model. The characteristic residence times for transient (τ_1_ ≈ 0.11 s) and stable (τ_2_ ≈ 0.22 s) dwell events were indistinguishable between variants. In contrast, removal of the extended N-terminal region significantly increased the probability of entering the stable immobilized state. Specifically, sFtsK-HT exhibited an approximately 36% relative increase in the stable dwell fraction (τ_2_), rising from 14.3% to 19.5% (*P* = 5.03 × 10^−8^; Fig. S5B). These results indicate that loss of the extended N-terminal region increases the frequency of stable immobilization events without altering the intrinsic stability of each state, suggesting more frequent but spatially non-productive confinement events in sFtsK-HT.

Consistent with these findings, spatial mapping of diffusion clusters revealed pronounced differences in localization patterns between the two variants (Fig. 3C). The K-means clustering applied to averaged MSD values resolved two dominant populations. The slow/confined population (cluster 1; ~93.55% of tracks) of full-length FtsK-HT (D_xy_ = 0.136 µm²/s; sub-diffusive) displayed strong mid-cell enrichment, consistent with productive confinement at the division septum. In contrast, the equivalent population of sFtsK-HT (93.68% of tracks; D_xy_ = 3.18 × 10^−2^ µm²/s; Brownian, *P* = 0.25) showed a diffuse distribution, lacking mid-cell enrichment. The fast mobile population (cluster 2, ~6.45% and 6.32% of tracks in FtsK-HT and sFtsK-HT, respectively) displayed Brownian motion in both variants; however, FtsK-HT trajectories retained preferential mid-cell enrichment, consistent with transient sampling of the septal region even among highly mobile molecules.

Collectively, these results demonstrate that the extended N-terminal region of FtsK is essential for promoting spatial clustering and constrained dynamics at the division site. Although its removal does not abolish septal localization, it shifts FtsK dynamics toward slower, more delocalized diffusion and increases non-productive immobilization events. We propose that the extended N-terminal region fine-tunes FtsK confinement by limiting off-target interactions, potentially with the membrane or non-divisome proteins, thereby ensuring high-fidelity localization at the divisome.

### The N-terminal region of FtsK mediates specific protein and lipid interactions

Single-particle tracking revealed that the removal of the extended N-terminal region of FtsK reduces diffusivity across all motile subpopulations and leads to a loss of sub-diffusive, septal-confined motion. Importantly, this defect does not abolish septal complex formation by sFtsK, indicating that the N-terminal region is not essential for core divisome recruitment. Instead, the altered dynamics suggested that the N-terminal region (nFtsK) may modulate interactions with “secondary” partners or suppress non-specific associations.

**Fig 4:**
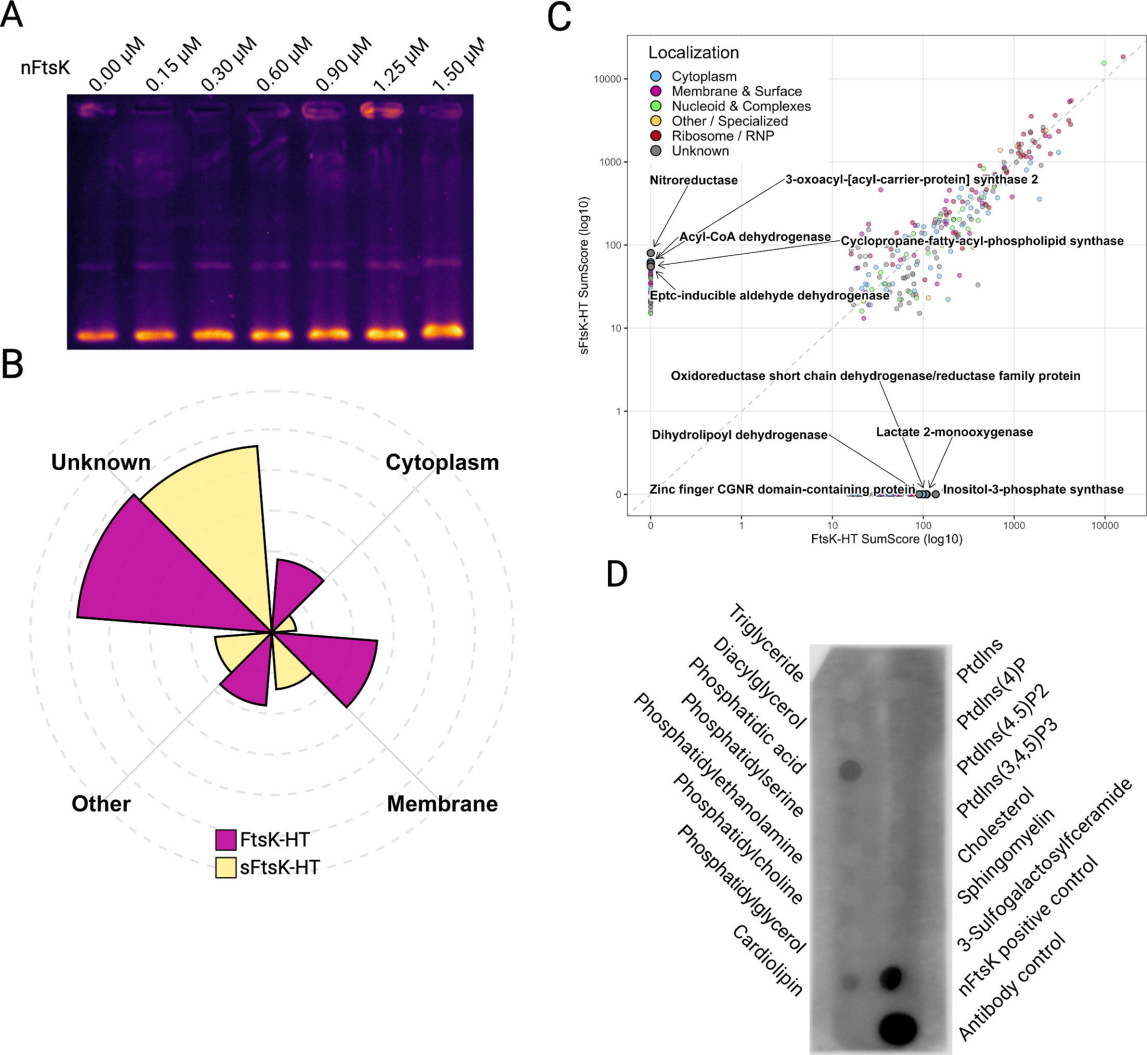
Functional characterization and interactome profiling of the N-terminal region of FtsK (nFtsK). (**A**) EMSA assessing the DNA-binding activity of purified nFtsK, showing no protein-DNA interaction. (**B**) Subcellular localization profiling of proteins identified exclusively in FtsK-HT or sFtsK-HT pulldowns. Functional categories were assigned based on UniProt “Subcellular location [CC]” annotations and transmembrane domain predictions. (**C**) Comparative scatter plot of the FtsK-HT and sFtsK-HT interactomes. Axes represent log_10_-transformed enrichment intensities. Labeled points indicate the top unique interactors of the full-length (FtsK-HT) and truncated (sFtsK-HT) variants, highlighting distinct interaction landscapes. (**D**) Lipid-protein overlay assay demonstrating the specificity of purified nFtsK-FLAG for phosphatidic acid and cardiolipin.

Given the net positive charge of nFtsK, we first tested whether this region mediates DNA binding. However, electrophoretic mobility shift assay (EMSA) revealed no detectable interaction between purified nFtsK (Fig. S6) and DNA, as no protein-DNA complexes were observed under the conditions tested (Fig. 4A). These data indicate that nFtsK does not function as an autonomous DNA-binding module.

We next examined whether nFtsK contributes to specific protein-protein interactions by comparing the interactome of FtsK-HT and sFtsK-HT (Fig. S7 and Table S1). Both variants co-purified robustly with established septal and divisome-associated proteins, including MSMEG_4287 (a recently identified septal factor), MSMEG_5223, SecA1, Ffh, and OxaA, the latter three being involved in membrane protein targeting. FtsK itself, as well as numerous proteins previously identified in FtsQ pulldowns, a close divisome partner, were also detected (22). Importantly, SepF, a membrane anchor for the Z-ring, was present in both data sets. Additionally, the nucleoid-associated proteins HupB and mIHF, as well as the chaperones GroEL2 and GroES, were detected in both interactomes. Together, these findings indicate that truncation of the N-terminal region does not disrupt recruitment of core divisome components, consistent with the preserved septal localization observed for sFtsK.

Despite this overall similarity, comparative analysis revealed a distinct set of proteins that were selectively enriched in the FtsK-HT interactome, implicating nFtsK in additional regulatory interactions (Fig. 4B and C). Notably, Ino1, the rate-limiting enzyme of inositol biosynthesis, and a key contributor to phosphatidylinositol-derived cell envelope components (phosphatidylinositol mannosides PIMs, lipoarabinomannan LAM, and lipomannan LM) was detected exclusively in the FtsK-HT pulldown. Several enzymes involved in acetyl-CoA production, critical for mycolic acid and peptidoglycan synthesis, were also uniquely associated with FtsK-HT, including two components of the pyruvate dehydrogenase complex and lactate 2-monooxygenase, which converts lactate to acetate, a direct precursor of acetyl-CoA. In addition, MSMEG_1813, required for lipid branching reactions, and WhiA, a known coordinator of cell division, were identified only in the presence of full-length FtsK. These associations suggest that nFtsK links septal translocation with metabolic pathways essential for cell envelope biogenesis.

In contrast, the sFtsK-HT interactome was characterized by increased enrichment of proteins indicative of aberrant or promiscuous interactions. The membrane protease FtsH, which targets misfolded or damaged membrane-associated proteins, was markedly enriched in the sFtsK-HT pulldown, exhibiting 13-fold higher enrichment compared to FtsK-HT. This suggests that removal of the terminal region renders FtsK more susceptible to quality control surveillance. Additionally, the δ subunit of DNA Polymerase III (a replisome component) was detected exclusively in the sFtsK-HT fraction. Ribosomal proteins, commonly associated with non-specific binding in pulldown experiments, were also enriched in the truncated variant. These observations are consistent with the increased non-specific immobilization and delocalized diffusion detected for sFtsK *in vivo*.

Finally, we examined whether nFtsK interacts with membrane lipids. Lipid-binding assays revealed that nFtsK preferentially associates with phosphatidic acid (PA) and cardiolipin (CL), two canonical lipids that accumulate in regions of negative membrane curvature (23–25), such as the septum and cell poles (Fig. 4D).

Collectively, these results demonstrate that the N-terminal region of FtsK engages in specific protein-protein and protein-lipid interactions. By associating with negatively curved membrane domains and metabolic regulators of cell envelope synthesis, nFtsK promotes spatial confinement and suppresses non-specific interactions, thereby stabilizing FtsK positioning and dynamics at the division site.

## DISCUSSION

Unlike laterally growing, symmetrically dividing model bacteria, mycobacteria elongate from the cell poles and divide asymmetrically (3, 4, 26) in the absence of the canonical MinCDE and nucleoid occlusion systems that ensure accurate septum placement and protect the chromosome. Asymmetric division produces daughter cells with distinct sizes and growth capacities, generating phenotypic heterogeneity within the population (3, 4, 26). Such heterogeneity is thought to act as a bet-hedging strategy that promotes survival under stress, including antibiotic challenge (4). Despite the lack of these spatial regulators, septum formation and closure occur with high fidelity, implying the existence of alternative mechanisms that couple divisome activity to chromosome dynamics. This raises the fundamental question of how late-stage chromosome segregation is coordinated with septum constriction to prevent guillotining of the chromosome and ensure genome integrity.

In mycobacteria, the essential DNA translocase FtsK (9, 10, 19) functions during the late stages of chromosome segregation, as in other bacteria. FtsK is recruited only after assembly of the core divisome, coinciding with the onset of septal constriction, and localizes precisely to the leading edge of the invaginating septum, forming a septal-like structure that ensures DNA is cleared before final scission (Fig. 2A). Unlike its homologs in symmetrically dividing bacteria, mycobacterial FtsK possesses an extended N-terminal region (Fig. 1A) that is conserved across both pathogenic and saprophytic mycobacterial species (Fig. 1B) and contains a conserved consensus sequence (Fig. 1C). This extension likely represents a mechanistic adaptation to the asymmetric mode of division, mediating specific interaction with potential partners, such as protein(s) and/or membrane lipid(s), to stabilize FtsK positioning and coordinate septal dynamics with chromosome translocation in the absence of canonical spatial regulators.

Removal of the N-terminal extension (nFtsK) results in a modest but reproducible decrease in cell length and a shorter generation time (Fig. 2D), without affecting septal recruitment, septum formation, or completion of division, indicating that the core divisome remains functional. Protein steady-state levels were comparable between strains (Fig. S3), excluding the possibility that the observed phenotypes reflect reduced abundance or instability of the truncated variant. Our data support a model in which nFtsK stabilizes late-stage divisome dynamics and transiently delays cytokinesis (Fig. 2D). In its absence, the cell cycle progresses more rapidly but with reduced temporal precision. Although the average timing of chromosome segregation relative to FtsK disassembly is preserved, the variability of this coordination is significantly increased in the sFtsK strain. Importantly, this increased temporal variability does not translate into overt fitness costs under standard laboratory conditions, as neither population viability nor the proportion of anucleate or dead cells differed significantly between strains (Fig. S4B). Thus, nFtsK does not alter the sequence of cell cycle events but constrains fluctuations around an optimal timing window, enhancing the robustness and fidelity of division rather than its speed.

The spatial organization of FtsK is strongly influenced by its N-terminal extension. sFtsK localizes closer to midcell than the full-length protein (Fig. 2C), indicating that the extension contributes to positioning FtsK at the non-central septa characteristic of mycobacteria. While both old and new pole daughter cells were consistently shorter in the sFtsK background, the asymmetry ratio between daughter cell lengths was not significantly altered (Fig. S2C). The uniform reduction in cell length likely reflects the shortened generation time in the sFtsK-HT strain (Fig. 2D), though the reduced septal residence time may also limit the window available for proper assembly of the late divisome and elongation machinery. The increased variance in septal signal positioning observed in longer sFtsK-HT cells further suggests a role for nFtsK in guiding septum placement with spatial precision (Fig. 2C, lower panel and Fig. S2B)—a function with potential implications for population heterogeneity under stress conditions.

Strikingly, the dominant confined population of sFtsK-HT loses mid-cell enrichment (Fig. 3C), consistent with the reduced overall diffusion observed across all mobility states (Fig. 3A and B and Fig. S5A). Dwell time analysis further shows that sFtsK-HT undergoes stable immobilization more frequently without a corresponding increase in septal anchoring, consistent with non-productive off-target confinement (Fig. S5B). Without nFtsK, FtsK retains septal recruitment but loses spatial confinement at the division site. We propose that this confinement—rather than mere septal localization—is required for robust coordination of septal synthesis with the replication cycle, consistent with the increased cell-to-cell variability in TMR-Trehalose incorporation timing and replication initiation in daughter cells (Fig. 2D).

Changes in FtsK dynamics are accompanied by altered protein interactions (Fig. 4B and C and Table S1). Despite truncation of the N-terminal region, approximately 70% of the combined interactome was shared between both variants (Fig. S7), and core divisome components associate with both full-length and truncated FtsK, consistent with preserved septal recruitment. However, sFtsK shows increased association with proteins linked to non-specific or aberrant interactions. Enrichment of FtsH in the sFtsK-HT pulldown suggests that, in the absence of nFtsK, FtsK may be more susceptible to quality-control surveillance, potentially due to inappropriate membrane contacts or altered interaction specificity. Full-length FtsK, by contrast, specifically associates with WhiA, a known division regulator, as well as enzymes involved in acetyl-CoA production and lipid biosynthesis, including Ino1. These associations suggest that nFtsK presumably couples late chromosome segregation to regulatory and metabolic processes required for septum maturation and new pole establishment.

Our data further indicate that nFtsK acts primarily through protein-lipid interactions rather than direct DNA binding. Despite its strong positive charge, nFtsK does not bind DNA *in vitro* (Fig. 4A). Instead, it preferentially associates with cardiolipin and phosphatidic acid (Fig. 4D), lipids enriched at regions of negative membrane curvature, such as the septum and cell poles. These observations are consistent with a role for lipid interactions in contributing to FtsK septal positioning, though the precise mechanistic relationship between nFtsK lipid binding and the dynamics observed *in vivo* remains to be established.

In summary, the N-terminal extension of mycobacterial FtsK acts as a lineage-specific regulatory module that fine-tunes the spatial and temporal execution of cytokinesis during asymmetric cell division. Although dispensable for division under standard laboratory conditions, nFtsK constrains cell-to-cell variability in divisome dynamics, septum positioning, and the coupling of cytokinesis to DNA replication and cell wall synthesis—properties likely important for fitness in stressful environments. Our findings suggest that mycobacterial FtsK has acquired a regulatory extension that adapts divisome function to the demands of asymmetric, polar growth, a mode of division that lacks the canonical spatial regulators present in model organisms.

## MATERIALS AND METHODS

### Plasmid propagation and bacterial cultivation

Plasmids used for *Mycobacterium smegmatis* mc^2^ 155 transformation were propagated in *Escherichia coli* DH5α. *E. coli* was cultured at 37°C in Luria-Bertani (LB) broth with shaking at 180 rpm or on LB agar plates (Difco). Media were supplemented with antibiotics (100 µg/mL ampicillin or 50 µg/mL kanamycin) and additives, such as 0.004% X-Gal when necessary (27). *M. smegmatis* liquid cultures were grown at 37°C with agitation at 180 rpm in Middlebrook 7H9 broth supplemented with 0.05% Tween 80 and albumin-dextrose-catalase (ADC; BD), or in Difco Nutrient Broth (NB; BD). For solid media, *M. smegmatis* was grown on NB supplemented with 2% agar or on Middlebrook 7H10 agar with oleic acid-albumin-dextrose-catalase (OADC; BD). Cultures were incubated at 37°C for 2–5 days until visible colonies appeared. If required, media were supplemented with 100 µg/mL ampicillin, 50 µg/mL kanamycin, 0.004% X-Gal, 2% sucrose, or 1 mM IPTG.

### Bacterial staining and sample preparation

For imaging, exponential-phase cultures of *M. smegmatis* grown in 7H9 supplemented with ADC and 0.05% Tween 80 (OD600 = 0.8–1.2) were harvested by centrifugation (5,000 × *g*, 5 min, room temperature), washed once with PBS, and resuspended in PBS. Exponential-phase cells were used for staining. For cell membrane visualization, log-phase cells were stained with 0.5 µM FM5-95 dye (Thermo Fisher Scientific). For HaloTag labeling in *M. smegmatis* (FtsK-HT and sFtsK-HT), TMRDirect ligand (Promega, 100 µM stock) was added at a 1:1,000 dilution to a final concentration of 100 nM. In all cases, 1 mL of the cell suspension was incubated with the respective dye for 30 min at 37°C with continuous agitation at 180 rpm. Following incubation, cells were centrifuged (5,000 × *g*, 5 min, RT), washed, and resuspended in PBS.

### Fluorescence microscopy

Samples (*M. smegmatis*) were mounted on Teflon-coated glass slides with 1.2% agarose. Snapshot analysis was performed using a Leica DM6 epifluorescence microscope equipped with an HC PL FLUOTAR 100×/1.32 OIL PH3 objective. The following imaging parameters were applied for FM5-95 (Thermo Fisher Scientific) and TMR Direct (Promega): excitation 570–590 nm, emission 602–662 nm; exposure time 300 ms; excitation intensity 40%. For NADA: excitation 450–490 nm, emission 500–550 nm; exposure time 100 ms; excitation intensity 32%. Images were processed and analyzed using Fiji software (28) and R software with the ggplot2 package (29).

### Time-lapse fluorescence microscopy

The *Mycobacterium smegmatis* liquid cultures grown overnight (OD600 ≈ 0.5) were loaded into an ONIX microfluidic chamber. Specifically, 70 µL of the cell suspension was injected into the inlet of a B04A plate (Merck). Prior to loading, the initial two wells were primed with PBS, whereas the subsequent wells contained 150–300 µL of 7H9 medium enriched with ADC and 0.05% Tween 80. After trapping the bacteria in the imaging chamber, a 45-min wash was performed with fresh medium at a pressure of 3 psi, followed by continuous cultivation for 24 h at 1.5 psi. For NADA-Green and 6-TMR-Trehalose (Tocris Bioscience) pulse-chase labeling, a 2-min labeling pulse was applied every 60 min. Where indicated, HaloTag fusions were labeled with either TMR Direct or R110 ligand (Promega). Microscopy data were collected using phase contrast and fluorescence channels (475/28 nm excitation/525/48 nm emission for NADA-Green and R110 Direct; 575/25 nm excitation/625/45 nm emission for TMR Direct and TMR-trehalose). The acquisition parameters included exposure times of 50–100 ms with 50% light intensity for the TMR Direct and 6-TMR-Trehalose signals, and 80–200 ms with 32% intensity for NADA-Green and R110. Automated time-lapse imaging was conducted at 10-min intervals on a DeltaVision Elite inverted microscope featuring a UPlanFL N 100×/1.3 Oil Ph3 objective and a temperature-controlled chamber set at 37°C. Post-acquisition analysis was performed in Fiji (28), and data visualization was generated using the ggplot2 package in R (R Foundation for Statistical Computing, Austria).

### Single particle tracking

Cells were cultured to mid-log phase in rich medium (7H9 supplemented with 10% ADC, 12.5 nM TMRDirect, and 0.05% Tween 80). Slides and coverslips were prepared by overnight incubation in 1 M KOH, followed by a milli-Q water wash and drying with pressurized nitrogen. Prior to imaging, cells were spread onto agar pads (1% low melting agarose in 7H9 poured into 1.0 × 1.0 cm GeneFrames) and sealed with clean 0.17-mm coverslips. Imaging was performed using a Zeiss Elyra 7 inverted microscope equipped with an sCMOS 4.2 CL HS camera and an alpha Plan-Apochromat 63×/1.46 Oil objective. The Z-axis was maintained via the Definite Focus.2 system. Data were recorded at 37°C using a 20 ms exposure per frame over 10,000 frames. For (s)FtsK-HaloTag strain stained with TMRdirect, a 561 nm laser was used at 30% intensity in TIRF mode (62° angle) with 500 mW power. For SPT analysis, spots were detected and reconstructed into tracks using the TrackMate v6.0.1 plugin in Fiji (30). Spot identification utilized a 0.5 μm diameter with sub-pixel localization, a median filter, and a signal-to-noise threshold of 5. Track reconstruction for FtsK-HT and sFtsK-HT allowed a maximum linking distance of 0.7 μm with no frame gaps; only tracks exceeding four frames were included in the final analysis. Statistical processing and comparison of the trajectories were conducted in SMTracker 2.0 (31). All analyses were performed on the same trajectory data set (FtsK-HT: *N* = 9,951 tracks; sFtsK-HT: *N* = 8,843 tracks). Dwell time was calculated using the stationary localization analysis module with a 100 nm confinement radius and a minimum of four consecutive steps, and was fitted with a two-component exponential decay model, yielding characteristic residence times τ₁ and τ₂. MSD (Mean Squared Displacement) was derived from the first five time points and fitted to a linear equation to determine the ensemble-averaged diffusion coefficient (D_xy_) and the anomalous diffusion exponent α; Brownian versus anomalous motion was assessed using an F-test for nested models as implemented in SMTracker 2.0. SQD (Squared Displacement) analysis used the cumulative distribution function of squared displacements to resolve up to three diffusive subpopulations using non-simultaneous independent fitting for each condition; statistical comparison of jump distance distributions between FtsK-HT and sFtsK-HT was performed using the Kolmogorov-Smirnov test. Spatial localization of diffusive subpopulations was assessed using the Clustering module, which applies a k-means algorithm to time-averaged MSD values of individual trajectories to classify tracks into groups of distinct dynamic behavior and to map their probability density of localization onto a normalized cell contour.

### Purification of His-nFtsK-FLAG

The obtained pellet of *E. coli* BL21 (DE3) pET28 nFtsK was resuspended in purification buffer A (PBS 10 mM imidazole), supplemented with 50 μL of viscolase (A&A Biotechnology). The cell suspension was sonicated using a Sonics Vibracell sonicator for 15 min at 40% amplitude, with a pulse cycle of 5 s on and 5 s off at 4°C. The resulting lysate was transferred to 50 mL Falcon tubes and centrifuged in a Beckman Avanti centrifuge for 40 min at maximum speed. The collected supernatant was filtered through a 0.22 μm syringe filter. The filtered supernatant was loaded onto a GE Healthcare ÄKTA chromatography system using HisTrap nickel columns. Proteins specifically bound to the resin were eluted using 30 mL of a linear gradient of elution buffer PBS 1.5 M imidazole. Remaining protein bound to resin was eluted with HEPES 20 mM, 500 mM imidazole, 500 mM NaCl, pH 7.2. The resulting protein preparations were analyzed by SDS-PAGE electrophoresis and western blot.

### Electrophoretic mobility shift assay

The EMSA was performed using purified His-nFtsK-FLAG protein to analyze DNA-protein interactions. A constant concentration of DNA (50–100 nmol per sample) was incubated with increasing concentrations of nFtsK (0.15, 0.3, 0.6, 0.9, 1.25, and 1.5 μM) for 20 min at room temperature. Subsequently, the samples were resolved on a 1% agarose gel. Electrophoresis was carried out overnight at 4°C to maintain complex stability. The following day, the gel was stained in TBE buffer supplemented with ethidium bromide for 30 min. DNA bands were visualized using a UV transilluminator and captured with a UV-sensitive camera system.

### Lipid-protein interaction assay

Lipid-protein interaction assays were performed using membrane lipid strips (Echelon Biosciences, P-6002) according to the manufacturer’s instructions. Briefly, 2 µL of a goat anti-mouse IgG secondary antibody conjugated to HRP (Invitrogen) and 2 µL of the His-nFtsK-FLAG protein were spotted onto the membrane as positive controls. The membrane was blocked overnight at 4°C with 5% non-fat dry milk in TBST (TBS + 0.1% Tween-20). The following day, the lipid strip was incubated for 1 h with 13 µg of His-nFtsK-FLAG protein in TBST buffer. After washing with TBST, the strip was incubated for 1 h with an anti-FLAG primary antibody (Sigma, F1804, 1:1,000 dilution). Following further washes with TBST, the membrane was incubated with an HRP-conjugated secondary antibody (Invitrogen, 1:5,000 dilution) for 1 h. Protein-lipid interactions were visualized using Pierce SuperSignal West Pico PLUS Chemiluminescent Substrate (Thermo Scientific) and detected with a ChemiDoc MP imaging system (Bio-Rad).

### Liquid chromatography-mass spectrometry sample preparation and analysis

Liquid chromatography-mass spectrometry (LC-MS) was performed on an M-Class Acquity UPLC connected to a Synapt XS HDMS equipped with a nanoESI source. Mobile phase A consisted of H_2_O + 0.1% FA, while mobile phase B consisted of ACN + 0.1% FA. An 8%–40% B 40 min linear gradient at a 300 nL/min flow rate was applied for sample separation on a C18 75 μm × 250 mm analytical column kept at 45°C. A 5-min sample trapping step was performed prior to analytical column separation. Data were collected in ESI+ using ion mobility DDA (HDDDA), with MS scan rate of 0.6 s, and MS/MS scan rate of 0.2 s, both in the 50–2,000 m/z range. The top eight precursors from each MS scan with charges 2+, 3+, and 4 + were selected for MS/MS, with one scan allowed per transition. A collision energy ramp determined specifically for each m/z (start LM, HM: 20–52 V; end LM, HM: 27–62 V) was applied on the transfer cell. Fragments not matching the precursor’s drift time were stripped. Source and IMS conditions were fine-tuned. A (Glu1)-Fibrinopeptide B solution was acquired in parallel as lockmass, and correction was applied post-acquisition. Four independent biological replicates were analyzed (*n* = 4), eight samples in total. Raw processing was performed using PLGS v3.0.3 (32). Obtained spectra were exported as .mgf files and searched using Mascot Server v2.8.0.1 (33) against the *Mycolicibacterium smegmatis (*formerly *Mycobacterium smegmatis)* protein sequence databank (UP000000757) to which porcine trypsin, human keratins, rat Ig gamma-2A C, and Ig lambda-2 C sequences were appended (34). Rat Ig sequences were identified in a SwissProt databank pre-search and are most likely resin-related. The search parameters were peptide mass tolerance: 15 ppm; fragment mass tolerance: 25 ppm; max. protein mass: 1 MDa; digest enzyme: trypsin; max. missed cleavages: 3; variable modification: oxidation of methionine; FDR (PSM): 1%. Protein hits were grouped into families. The Mascot protein hit .csv files were exported for each search, and all families were combined into a single list, along with their score sum, as well as peptide and occurrence counts for either FtsK-HT or sFtsK-HT, separately. Proteins detected across all replicates were retained, yielding a combined list of 320 proteins. Proteins were classified as “FtsK-HT unique” (*n* = 55) or “sFtsK-HT unique” (*n* = 40) if their HitCount was equal to zero in the opposing pull-down condition. Proteins detected in both pull-downs were classified as “Shared” (*n* = 225). The complete annotated data set is provided in Table S1. The mass spectrometry proteomics data have been deposited to the ProteomeXchange Consortium via the PRIDE (35) partner repository with the data set identifier PXDPXD073126.

### Phylogenetic analysis of Ftsk and conservation of the nFtsk region across *Mycobacteriaceae*

FtsK protein sequences were retrieved by BLASTp search (NCBIWWW, Biopython; E-value ≤ 1 × 10⁻⁵, BLOSUM62 matrix) against the NCBI non-redundant protein database, using *M. smegmatis* mc² 155 FtsK (ABK76147.1; 926 aa) as query. The search was restricted to *Mycobacterium*, *Mycolicibacterium*, *Mycobacteroides*, *Mycolicibacter*, and *Mycolicibacillus* genera, with *Streptomyces coelicolor* A3(2) included as outgroup. One representative sequence per species was retained, prioritizing type strains and RefSeq accessions (WP_/NP_). The final data set comprised 226 sequences: 107 slow-growing mycobacteria (*Mycobacterium sensu stricto*), 118 fast-growing mycobacteria (*Mycolicibacterium*, *Mycobacteroides*, *Mycolicibacter*, *Mycolicibacillus*), and 1 outgroup. Sequences were aligned using MAFFT v7.526 (--auto; FFT-NS-2 algorithm), yielding an alignment of 226 sequences × 1,407 positions (36). A maximum-likelihood phylogenetic tree was constructed using IQ-TREE 2 (v2.3.6) with automatic model selection by ModelFinder (BIC criterion), yielding the Q.insect+F+I+G4 substitution model (37, 38). Node support was assessed by 1,000 ultrafast bootstrap replicates (UFBoot2) and 1,000 SH-aLRT replicates; branches with SH-aLRT ≥ 80% and UFBoot ≥ 95% were considered well-supported (39). *S. coelicolor A3(2)* (QFI45605.1) was specified as the outgroup. The tree was visualized and annotated in iTOL (Interactive Tree Of Life), with slow and fast growers distinguished by colored range annotations (40). The nFtsK region was defined as the N-terminal sequence of FtsK preceding the first transmembrane helix (TM1), identified by Kyte-Doolittle hydrophobicity analysis (41) (sliding window = 19 aa) of *M. smegmatis* mc² 155 FtsK (ABK76147.1), corresponding to residues 1–151 (alignment position 288 of the MSA). nFtsK sequences were extracted from the MSA for 225 sequences (outgroup excluded). Sequence logos were generated using Logomaker v0.8 separately for slow-growing and fast-growing mycobacteria, with positions present in <15% of sequences omitted (42). The length of the nFtsK region was compared between slow and fast growers using the Wilcoxon rank-sum test. The full FtsK multiple sequence alignment and nFtsK region sequences alignment were deposited on Figshare and are available under https://doi.org/10.6084/m9.figshare.31915248.

### Statistical analysis and data visualization

Data were derived from three independent biological replicates for every investigated strain, unless otherwise stated in the figure legends or main text. The homogeneity of variance was verified using Levene’s test implemented in the *car* package within the RStudio environment (R Foundation for Statistical Computing, Austria). Based on the variance structure, statistical significance was determined using either the standard Student’s *t*-test or Welch’s *t*-test for unequal variances. Unless otherwise noted, numerical variations are reported as the standard deviation (SD), which is also represented by the error bars in the figures. Both statistical computations and graph generation were executed in R using the *stats* and *ggplot2* libraries (29). The thresholds for statistical significance were set as follows: *P* < 0.05 (*), *P <* 0.01 (**), *P <* 0.001 (***), and *P* < 0.0001 (****), while non-significant results are marked as “ns.” Schematics and model figures were designed using BioRender (BioRender.com).

## Data Availability

All data supporting the findings of this study are included in the article and its supplementary materials. The mass spectrometry proteomics data have been deposited in the ProteomeXchange Consortium via the PRIDE partner repository under the data set identifier PXD073126. The full FtsK multiple sequence alignment and nFtsK region sequence alignment generated in this study have been deposited in Figshare and are publicly available at https://doi.org/10.6084/m9.figshare.31915248. Further inquiries can be directed to the corresponding author.

## SUPPLEMENTAL MATERIAL

Supplemental materials jb.00096-26-s0001.pdf Supplemental Materials and Methods, Tables S2 to S4, Figures S1 to S7.

Table S1 jb.00096-26-s0002.docx Quantitative analysis of FtsK and sFtsK protein-protein interactions.

ASM does not own the copyrights to Supplemental Material that may be linked to, or accessed through, an article. The authors have granted ASM a non-exclusive, world-wide license to publish the Supplemental Material files. Please contact the corresponding author directly for reuse.
