# Supplementary material for: FtsK with a unique N-terminal extension is involved in coordinating the final steps of chromosome segregation with asymmetric division in mycobacterial cells
Source: J Bacteriol. 2026 May 29;208(6):e00096-26. doi: 10.1128/jb.00096-26 (PMC13277299; doi:10.1128/jb.00096-26)
Supplement: Supplemental materials — and Methods, Tables S2 to S4, Figures S1 to S7. [file jb.00096-26-s0001.pdf]

Material and Methods

Construction of *M. smegmatis mc*^{2} 155 mutant strains

The allelic replacement of *ftsk* gene (*MSMEG_2690*) with fusion genes *ftsk-halotag or sftsk-halotag* was performed following the protocol outlined by Parish and Roberts (1). Briefly, the chromosome of *M. smegmatis mc*^{2} *155* was used as template to amplify region of *ftsk* gene using one primer set FtsK_p2NIL_FW x FtsK_p2NIL_RV for FtsK-HT strain and two primer sets for sFtsK_F1_p2NIL_FW x sFtsK_F1_p2NIL_RV and sFtsK_F2_p2NIL_FW x sFtsK_F2_p2NIL_RV. In the case of *halotag* fusion gene, it was amplified by PCR utilizing primer sets – FtsK_HT_p2NIL_FW x FtsK_HT_p2NIL_RV. Amplified products were cloned to p2NIL Ø plasmid and transformed colonies were spread on 7H10+OADC medium supplemented with kanamycin. Sequenced vectors were used in transformation of *M. smegmatis*.

Construction *E. coli* BL21 (DE3) expressing nFtsK-mNeonGreen and microscopic analysis

To visualize sFtsK in *E. coli* cells, PCR-amplified products were generated using two sets of primers: nFtsK_pACYC_Fw x link_nFtsK_pACYC_Rv and nFtsK_link_mNG_pACYC_Fw x pACYC_mNG_NheI_Rv (**Table 2**). The resulting fragments were cloned into the pACYCDuet™-1 vector (Sigma) via Sequence and Ligation Independent Cloning (SLIC). Beforehand, the vector was linearized using the restriction enzymes NcoI-HF and HindIII-HF (NEB). For the negative control, mNeonGreen was PCR-amplified using the primer set pACYC_KN_Link_mNG_F x pACYC_mNG_NheI_Rv. Transformants were selected on LB agar supplemented with chloramphenicol. The resulting plasmids were verified by PCR and sequencing, and then transformed into *E. coli* BL21(DE3) cells. Positive clones were selected for fluorescence microscopy experiments.

SDS-PAGE and Western Blot

Western blotting analysis was performed to assess the expression levels and purity of the fusion proteins. The eluted fractions were resolved by SDS-PAGE using 8% or 10% gels. Following electrophoresis, gels were either stained with InstantBlue® Coomassie Protein Stain (Abcam, ab119211) or processed for Western blotting by transferring the proteins onto a nitrocellulose membrane. To prevent non-specific binding, the membrane was blocked overnight at 4°C with 5% non-fat dry milk in TBST (TBS + 0.1% Tween-20). The primary antibodies utilized were

anti-FLAG (Sigma, F1804, 1:1000 dilution) for His-nFtsK-FLAG and anti-6xHis (Invitrogen, MA1-135, 1:1000 dilution). Membranes were incubated with the primary antibody for 1 h at room temperature. After washing with TBST, the membranes were incubated for 1 h at room temperature with a goat anti-mouse IgG secondary antibody conjugated to HRP (Invitrogen, 1:5000 dilution). Protein bands were visualized using Pierce™ SuperSignal™ West Pico PLUS Chemiluminescent Substrate (Thermo Scientific) and captured with a ChemiDoc MP imaging system (Bio-Rad). Protein transfer and loading consistency were verified via Ponceau S staining (ThermoFisher Scientific) according to the manufacturer's instructions.

Pull-down assay

Liquid cultures of strains expressing FtsK-HT and sFtsK-HT were cultivated to an optical density (OD_{600}) of approximately 0.8. Biomass was harvested by centrifugation at 5,000 rpm for 20 minutes at 4°C, washed twice with ice-cold PBS, and resuspended in 10 mL of freshly prepared immunoprecipitation (IP) buffer (50 mM Tris-HCl pH 8.0, 250 mM NaCl, 0.8% Triton X-100) supplemented with a protease inhibitor cocktail (Pierce Protease Inhibitor, Thermo Scientific). Lysis was performed via sonication on ice for a total process time of 25 minutes (50% amplitude; 5-second pulse/5-second pause cycles). The resulting lysate was clarified by centrifugation at 10,000 rpm for 20 minutes at 4°C, followed by a second centrifugation step to ensure complete removal of cellular debris. Total protein concentration was quantified using the Bradford method (ROTI®Quant, Carl Roth). For the affinity purification, 10 mg of total protein lysate was diluted to a final volume of 14 mL with IP buffer. Magne HaloTag beads (100 µl; Promega) were equilibrated by washing three times in IP buffer to remove the storage solution, then added to the lysate and incubated overnight at 4°C with constant rotation. Following incubation, the beads were isolated using a magnetic rack and washed four times with 1 mL of cold IP buffer to remove non-specifically bound contaminants. To remove the detergent from the IP buffer, beads were washed four times in 25 mM Tris pH 7.5. Bead bound proteins were then denatured at 65°C for 10 minutes in 25 mM Tris pH 7.5, 0.1% sodium deoxycholate, 3mM DTT. Subsequently, 200 ng of trypsin was added to the sample for an overnight on-bead digestion in 37°C. Next day the solution was separated from the beads, acidified and sodium deoxycholate was removed by centrifugation. The supernatant was then desalted using a STAGE tip (2). Obtained peptide pellet was resuspended in 0.1% formic acid (FA), 3% acetonitrile (ACN) solution.

Supplementary tables

Table S2. Primers used in the study

**Name**	**Sequence**	**Application**
Ms_attB_L5_down	AGGCACATGCTGCCACTG	Strain construction
Ms_attB_L5_up	AGCGGATGCGCTACCAAG	
sFtsK_F1_FW	GCATTAAAGCTTCACGTGGTCGACGTTCACCCGGATGCTGTGCGGG	Construction of *p2NIL sftsK*
sFtsK_F1_RV	GCGCGCGGCCGACCAAGGAGCCGTGCGTACAT	
sFtsK_F2_FW	CACGGCTCCTTGGTCGGCCGCGCGCGT	
sFtsK_F2_RV	GGGAATTCTTAATTAAGCGGCCGCGGTACCGATGGCGTCGGTCATCTGGTC	
FtsK-HT_F1_FW	ATAAACTACCGCATTAAATCGTGGCGATCGTC	Construction of *p2NIL ftsK-halotag*
FtsK-HT_F1_RV	CGGTACCTTAACCGAACTCCTCGCCG	
FtsK-HT_F2_FW	GGAGTTCGGTTAAGGTACCGGCTCGGC	
FtsK-HT_F2_RV	CGGCAAGCTTAAGCTTTCAACCGGAAATCTCCAG	
FtsK-HT_F3_FW	TTGAAAGCTTAAGCTTGCCGAGAGTCCTAC	
FtsK-HT_F3_RV	TGACACTATAGAATACATAGGTGAACAGGAACACCAGGAAC	
nFtsK_pACYC_Fw	TAACTTTAATAAGGAGATATACATGTTGCTCATACAACGATCACTGG	Construction of *pACYCDuet™-1 nftsK-mNeonGreen*
link_nFtsK_pACYC_Rv	AGCCGACATAAGCTTGTGCCCGGGCTCGAT	
nFtsK_link_mNG_pACYC_Fw	ATCGAGCCCGGGCACAAGCTTATGTCGGCTGGCT	
pACYC_mNG_NheI_Rv	TCGACTTAAGCATTATGCGGCCGCAGCTAGCTTATTTGTACAATTCATCCATGCC	

pACYC_KN_Link_mNG_F	GTTTAACTTTAATAAGGAGATATACATGTCGGCTGGCTCCG	Construction of *pACYCDuet™-1 linker-mNeonGreen*
pACYC_mNG_NheI_Rv	TCGACTTAAGCATTATGCGGCCGCAGCTAGCTTATTTGTACAATTCATCCATGCC	
FtsK_N-term_FW	GGTACCTTGCTCATACAACGATCACTGGAC	Construction *pET28 nftsk-FLAG*
FtsK_N-term_RV	CTCGAGGTGCCCGGGCTCGATGTCA	

Table S3. Plasmids used in the study

Name	Plasmid feature	Reference
p2NIL Ø	kanamycin resistance, *oriE*, suicide plasmid for allelic replacement	(1)
pGOAL17 Ø	ampicillin resistance, *oriE*, selective PacI selective cassete with *lacZ*, *sacB* and *kanR* genes	(1)
pMV$_{pAMI}$ Ø	kanamycin resistance, *oriE*, inducible promotor *p$_{AMI}$, attB* integrative plasmid for mycobacterial transformation	Lab collection
p2NIL ftsK-halotag	plasmid constructed on p2NIL Ø backbone with inserted *ftsk-halotag*	This study
p2NIL ftsK-halotag GOAL	plasmid constructed on *p2NIL ftsK-halotag* backbone with inserted *goal* cassette from pGOAL17 Ø.	This study
p2NIL sftsK	plasmid constructed on p2NIL Ø backbone with inserted DNA sequence of FtsK without first 141 aminoacids	This study
p2NIL sftsK GOAL	plasmid constructed on *p2NIL sftsK* backbone with inserted *goal* cassette from pGOAL17 Ø.	This study
pMVpNAT DnaN-mCherry	plasmid constructed on pMV$_{306}$ Ø backbone with inserted promotor of DnaN and DnaN-mCherry	Lab collection

p2NIL HupB-mCherry GOAL	plasmid constructed on *p2NIL hupB-mCherry* backbone with inserted *goal* cassette from pGOAL17 Ø.	(3)
pACYCDuet™-1 Ø	CmR, *cat* promoter, p15A *ori*, *lac* promotor, repressor, operator, His-tag, S-tag	Lab collection
pACYCDuet™-1 nftsK-mNeonGreen	plasmid constructed on pACYCDuet™-1 Ø backbone with inserted *nftsK-mNeonGreen*	This study
pACYCDuet™-1 linker-mNeonGreen	plasmid constructed on pACYCDuet™-1 Ø backbone with inserted *linker-mNeonGreen*	This study
pET28a(+) Ø	kanamycin resistance, pBR322 origin, T7 promoter/expression system with N-terminal His-tag, thrombin cleavage site, and T7 tag. 3xFLAG sequence inserted af	Lab collection
pET28a nftsK-FLAGx3	plasmid constructed on pET28a(+) Ø backbone with inserted *nftK-FLAGx3*	This study

Table S4. Strains used in the study

**Name**	**Genotype**	**Source**
WT	*M. smegmatis mc^2 155*	Lab collection
FtsK-HT	*M. smegmatis mc^2 155 ftsK-halotag*	This study
sFtsK-HT	*M. smegmatis mc^2 155 sftsK-halotag*	This study
FtsK-EGFP HupB-mCherry	*M. smegmatis mc^2 155 ftsK-egfp hupB-mCherry*	This study
FtsK-HT DnaN-mCherry	*M. smegmatis mc^2 155 ftsK-halotag attBL5::pMV306$_{pnat\ dnaN\text{-}mCherry}$*	This study
sFtsK-HT DnaN-mCherry	*M. smegmatis mc^2 155 sftsK-halotag attBL5::pMV306$_{pnat\ dnaN\text{-}mCherry}$*	This study
FtsK-HT HupB-mCherry	*M. smegmatis mc^2 155 ftsK-halotag attBL5::pMV306$_{pnat\ hupB\text{-}mCherry}$*	This study

sFtsK-HT HupB-mCherry	*M. smegmatis mc² 155 sftsK-halotag attBL5::pMV306$_{pnat\ hupB\text{-}mCherry}$*	This study
His-nFtsK-FLAG	*E. coli BL21 (DE3) pET28 His-nFtsK-FLAG*	This study
KN-mNG	*E. coli BL21 (DE3) pACYC linker-mNeonGreen*	This study
nFtsK-mNG	*E. coli BL21 (DE3) pACYC nFtsK-mNeonGreen*	This study

Supplementary figures

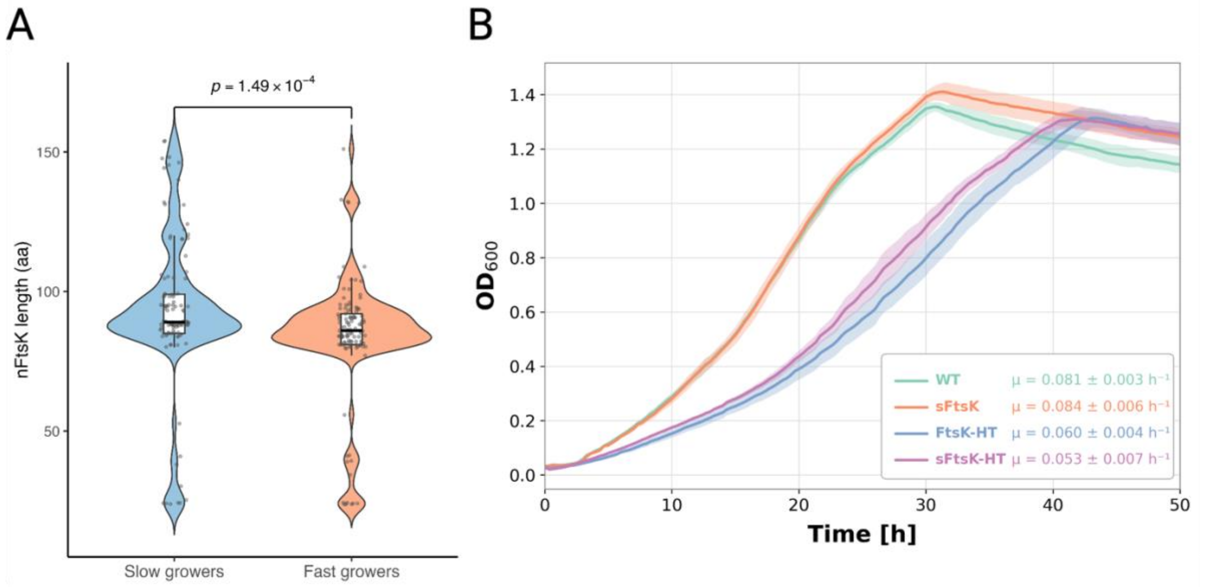

Fig. S1. Growth rates of *M. smegmatis* strains. (A) Violin plots showing the distribution of nFtsK fragment lengths (amino acids) in slow- and fast-growing mycobacterial species. The nFtsK fragment is significantly longer in slow growers than in fast growers ($p = 1.49 \times 10^{-4}$). **(B)** Growth curves of wild-type (WT) and FtsK derivative strains. Lines represent mean optical density at 600 nm (OD_{600}) over time; shaded areas indicate ± standard deviation (n = 6). Calculated exponential growth rates are indicated in the legend. HaloTag (HT) fusion strains exhibit reduced growth rates compared to their non-tagged counterparts (WT vs. FtsK-HT and sFtsK vs. sFtsK-HT). Introduction of the sFtsK version does not significantly affect growth rate compared to the respective parental strain (WT vs. sFtsK and FtsK-HT vs. sFtsK-HT; $p > 0.05$).

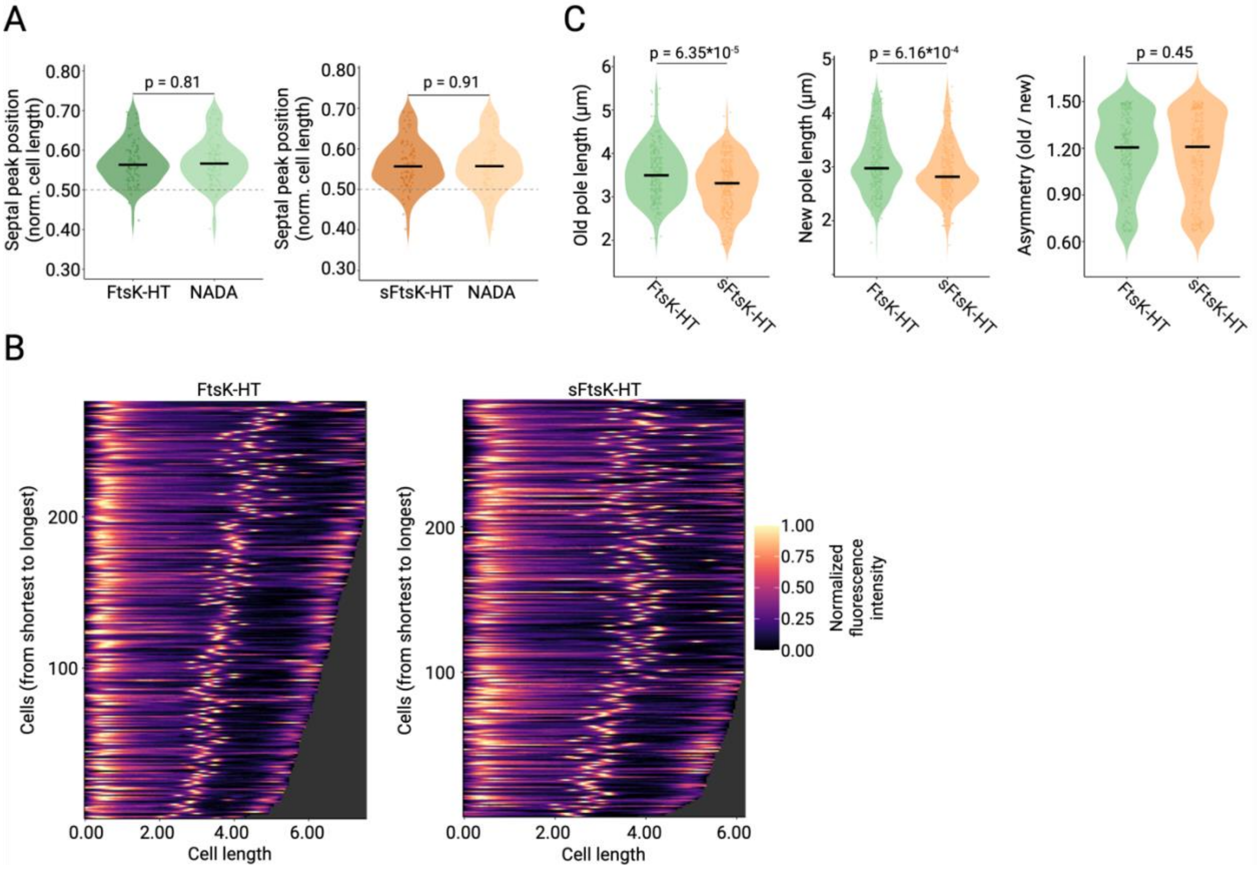

Figure S2. Subcellular localization and cell division asymmetry of FtsK-HT and sFtsK-HT. (A) Septal peak position (normalized cell length) for FtsK-HT (left, n = 87) and sFtsK-HT (right, n = 90) compared to septum stained with NADA, a fluorescent D-amino acid. Dashed line indicates the cell midpoint – 0.5. **(B)** Fluorescence intensity heatmaps for FtsK-HT (n = 276) and sFtsK-HT (n = 287), aligned to the old pole and sorted by cell length. Color scale represents per-cell normalized fluorescence intensity. **(C)** Old pole length, new pole length, and pole asymmetry ratio (old/new) for FtsK-HT and sFtsK-HT. FtsK-HT cells had a significantly longer old pole (3.51 ± 0.60 µm vs 3.28 ± 0.60 µm, $p = 6.40 \times 10^{-5}$) and new pole (3.05 ± 0.54 µm vs 2.91 ± 0.49 µm, $p = 6.20 \times 10^{-4}$) compared to sFtsK-HT, while the asymmetry ratio did not differ significantly between strains (p = 0.45; n = 276 and 287 for FtsK-HT and sFtsK-HT, respectively).

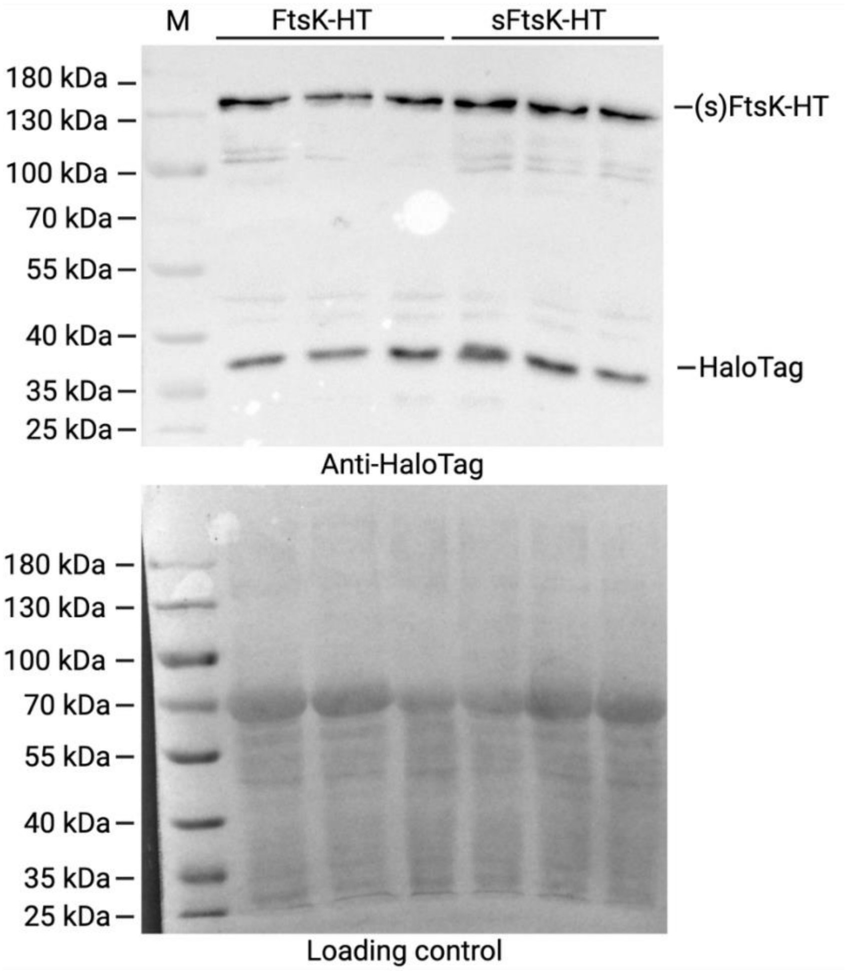

Figure S3. Steady-state abundance and proteolytic profile of FtsK-HT and sFtsK-HT. Western blot of whole-cell lysates from exponentially growing *M. smegmatis* strains probed with anti-HaloTag antibody. Both FtsK-HT (~147 kDa) and sFtsK-HT (~132 kDa) are detected at their expected molecular weights. A band at ~38 kDa, present in both strains at comparable intensity, is consistent with proteolytically released HaloTag, as previously described for HaloTag fusion proteins in bacteria. Additional intermediate bands observed between ~40 and 120 kDa are present in both strains and likely represent partial degradation products of (s)FtsK-HT.

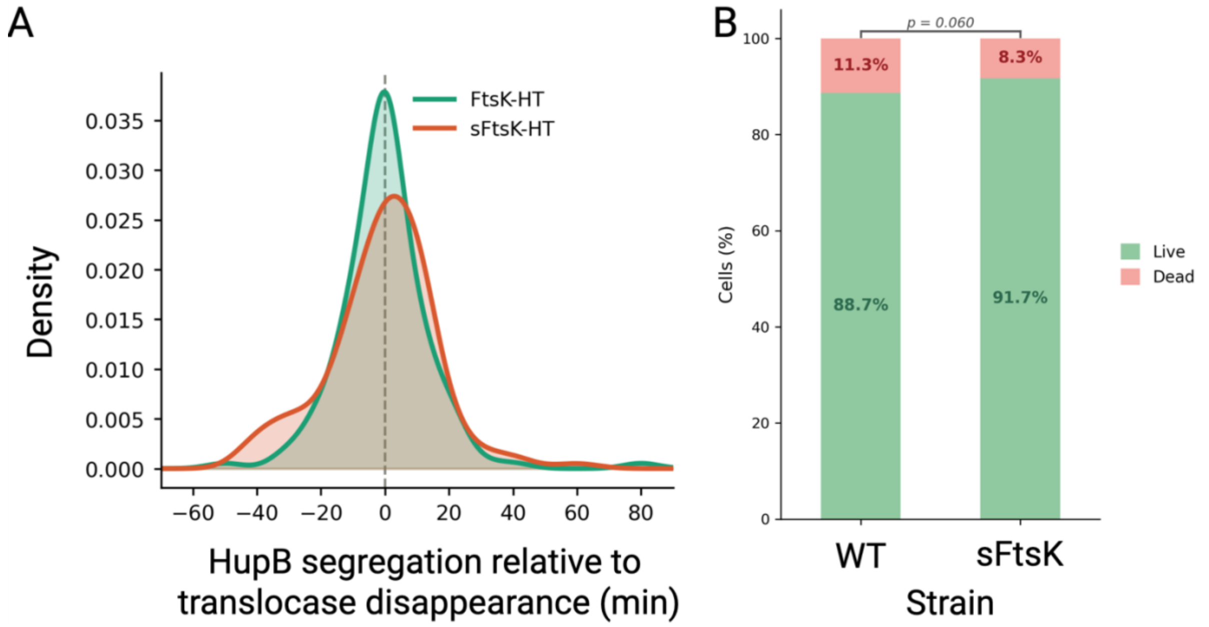

Figure S4. Chromosome segregation timing and cell viability in FtsK-HT and sFtsK-HT strains. (A) Density distributions of the time interval between translocase disassembly and HupB nucleoid splitting in FtsK-HT (green) and sFtsK-HT (orange) cells. The dashed line indicates translocase disappearance (t = 0 min); negative values denote segregation preceding, and positive values denote segregation following, translocation termination. Mean intervals were indistinguishable between strains (−0.1 ± 14.1 min for FtsK-HT vs. −0.9 ± 16.8 min for sFtsK-HT; Welch's t-test, p = 0.667), however cell-to-cell variability was significantly increased in sFtsK-HT cells (variance: 281 vs. 198; Levene's test, p = 0.023; n = 134 and 120 for FtsK-HT and sFtsK-HT, respectively). **(B)** Proportion of live and dead cells in exponentially growing cultures of WT and sFtsK strains assessed by live/dead fluorescence staining using SYTO 9 and propidium iodide (PI), marking DNA in live and dead cells, respectively. The dead fraction includes both PI-positive and anucleate cells. No significant difference in cell viability was detected between strains (chi^2 = 3.54, p = 0.060; n = 794 and 689 for WT and sFtsK, respectively).

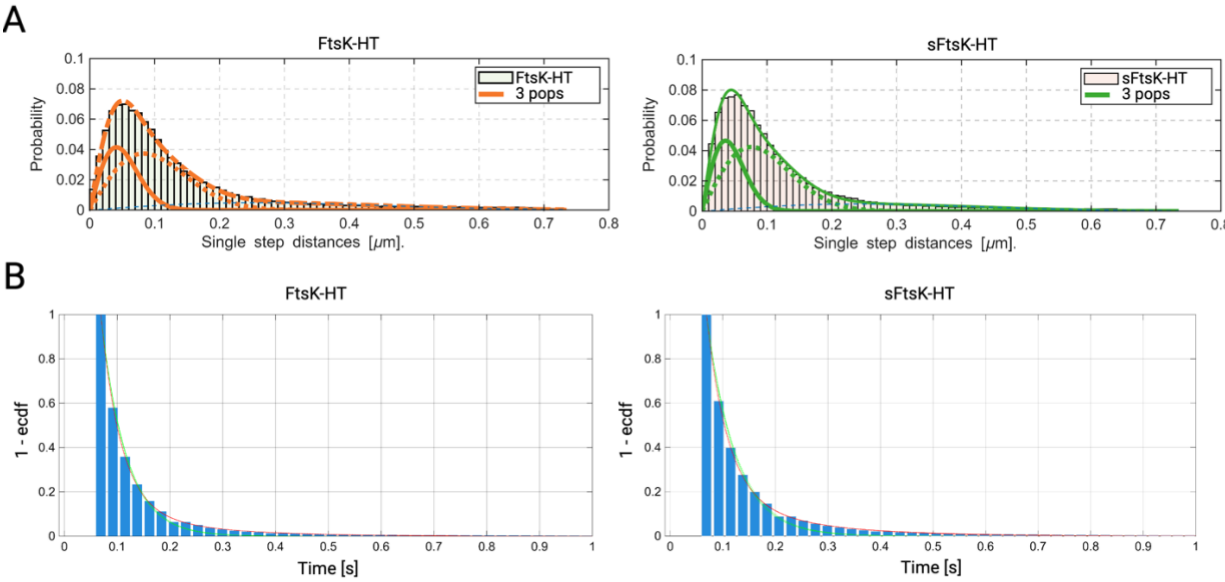

Figure S5. Single-step displacement distributions and dwell event analysis for FtsK-HT and sFtsK-HT. (A) Probability density histograms of single-step displacements for FtsK-HT (left) and sFtsK-HT (right), derived from Squared Displacement (SQD) analysis. Solid lines represent the best-fit model assuming three diffusive subpopulations. Individual subpopulation components are indicated as follows: confined (thick solid), slow mobile (dashed for FtsK-HT; thin solid for sFtsK-HT), and fast mobile (dotted). **(B)** Dwell time distributions are presented as survival functions (1 – eCDF, where the y-axis represents the probability that a dwell event has a duration longer than t) for FtsK-HT and sFtsK-HT (N =9951 and 8843 tracks, respectively), derived from Stationary Localization Analysis (SLA) with a confinement radius of 100 nm. Green line: single-component exponential fit (τ = 0.12 s for both variants); red line: two-component exponential fit resolving a transient ($\tau_1 \approx$ 0.11 s) and a stable ($\tau_2 \approx$ 0.22/0.23 s) dwell population. The fraction of molecules in the stable dwell state (τ_2) was significantly increased in sFtsK-HT relative to FtsK-HT (19.5 ± 2.5% vs. 14.3 ± 2.0%; $p = 5.03 \times 10^{-8}$).

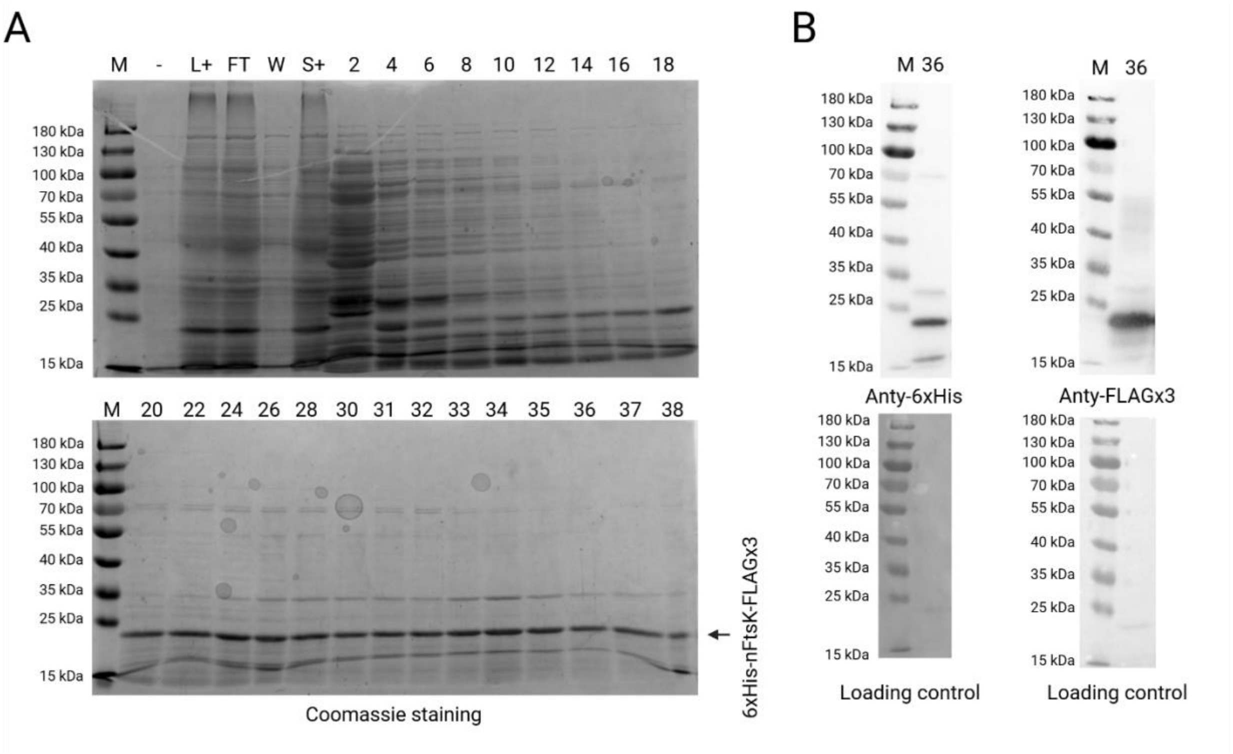

Figure S6. Purification and quality assessment of 6xHis-nFtsK-FLAG. A. SDS-PAGE analysis of eluted fractions from 6xHis-nFtsK-FLAG purification. Fraction 36 was selected for subsequent downstream analysis. The predicted molecular weight of 6xHis-nFtsK-FLAGx3 is 22.6 kDa. **B.** Western blot analysis of fraction 36 to confirm the identity of the target protein and determine whether additional bands represent degradation products or other species of the target protein. The primary band corresponds to the estimated molecular weight of 6xHis-nFtsK-FLAGx3 (22.6 kDa).

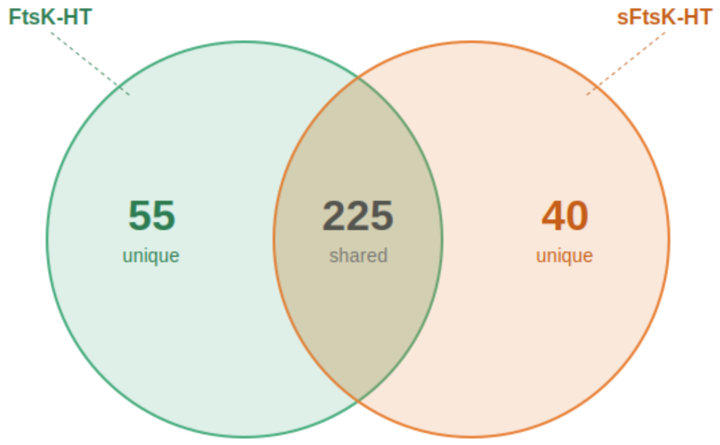

Figure S7. Overlap between FtsK-HT and sFtsK-HT interactomes in *M. smegmatis*. Venn diagram showing the number of proteins identified in pull-down experiments with FtsK-HT and sFtsK-HT. Numbers indicate proteins unique to each condition or shared between both. A total of 320 proteins were detected across both conditions, of which 225 (70.3%) were shared, 55 were exclusive to FtsK-HT, and 40 were exclusive to sFtsK-HT. The full list of obtained hits is available in Table S1

1. Parish T, Roberts DM. 2015. Mycobacteria protocols: Third edition Mycobacteria Protocols: Third Edition. Springer New York, New York, NY. http://www.springer.com/series/7651.

2. Rappsilber J, Ishihama Y, Mann M. 2003. Stop and Go Extraction Tips for Matrix-Assisted Laser Desorption/Ionization, Nanoelectrospray, and LC/MS Sample Pretreatment in Proteomics. Anal Chem 75:663–670.

3. Hołówka J, Trojanowski D, Ginda K, Wojtaś B, Gielniewski B, Jakimowicz D, Zakrzewska-Czerwińska J. 2017. HupB Is a Bacterial Nucleoid-Associated Protein with an Indispensable Eukaryotic-Like Tail. mBio 8.
