# Supplementary material for: FtsK with a unique N-terminal extension is involved in coordinating the final steps of chromosome segregation with asymmetric division in mycobacterial cells
Source: J Bacteriol. 2026 May 29;208(6):e00096-26. doi: 10.1128/jb.00096-26 (PMC13277299; doi:10.1128/jb.00096-26)
Supplement: Table S1 — Quantitative analysis of FtsK and sFtsK protein-protein interactions. [file jb.00096-26-s0002.docx]

| **Table S1. Quantitative analysis of FtsK-HT and sFtsK-HT protein-protein interactions.** | | | | | | | | | |
| --- | --- | --- | --- | --- | --- | --- | --- | --- | --- |
|  |  | **FtsK-HT** | | | **sFtsK-HT** | | |  |  |
| **Protein Accession** | **Gene Name(s)** | **Sum Score** | **Sum Peptides** | **Hit Count** | **Sum Score** | **Sum Peptides** | **Hit Count** | **Protein  Description** | **Category** |
| A0R7G6 | ino1 MSMEG_6904 MSMEI_6720 | 137 | 6 | 3 |  |  |  | Inositol-3-phosphate synthase OS=Mycolicibacterium smegmatis (strain ATCC 700084 / mc(2)155) OX=246196 GN=ino1 PE=1 SV=1 | **FtsK-HT unique** |
| A0QZB3 | MSMEG_3962 | 109 | 4 | 3 |  |  |  | Lactate 2-monooxygenase OS=Mycolicibacterium smegmatis (strain ATCC 700084 / mc(2)155) OX=246196 GN=MSMEG_3962 PE=3 SV=1 | **FtsK-HT unique** |
| A0QPE7 | MSMEG_0372 | 107 | 3 | 3 |  |  |  | Oxidoreductase short chain dehydrogenase/reductase family protein OS=Mycolicibacterium smegmatis (strain ATCC 700084 / mc(2)155) OX=246196 GN=MSMEG_0372 PE=1 SV=1 | **FtsK-HT unique** |
| A0QQW8 | lpdA MSMEG_0903 | 98 | 2 | 2 |  |  |  | Dihydrolipoyl dehydrogenase OS=Mycolicibacterium smegmatis (strain ATCC 700084 / mc(2)155) OX=246196 GN=lpdA PE=3 SV=1 | **FtsK-HT unique** |
| A0QNP6 | MSMEG_0118 | 90 | 4 | 3 |  |  |  | Zinc finger CGNR domain-containing protein OS=Mycolicibacterium smegmatis (strain ATCC 700084 / mc(2)155) OX=246196 GN=MSMEG_0118 PE=4 SV=1 | **FtsK-HT unique** |
| A0QSL4 | rpmJ MSMEG_1520 MSMEI_1484 | 78 | 2 | 2 |  |  |  | Large ribosomal subunit protein bL36 OS=Mycolicibacterium smegmatis (strain ATCC 700084 / mc(2)155) OX=246196 GN=rpmJ PE=1 SV=1 | **FtsK-HT unique** |
| A0QQ62 | MSMEG_0640 | 73 | 3 | 2 |  |  |  | Oligopeptide transport ATP-binding protein OppD OS=Mycolicibacterium smegmatis (strain ATCC 700084 / mc(2)155) OX=246196 GN=MSMEG_0640 PE=3 SV=1 | **FtsK-HT unique** |
| A0R1B3 | pepN MSMEG_4690 | 72 | 3 | 2 |  |  |  | Aminopeptidase N OS=Mycolicibacterium smegmatis (strain ATCC 700084 / mc(2)155) OX=246196 GN=pepN PE=3 SV=1 | **FtsK-HT unique** |
| A0QQ61 | MSMEG_0639 | 71 | 2 | 2 |  |  |  | Oligopeptide transport ATP-binding protein AppF OS=Mycolicibacterium smegmatis (strain ATCC 700084 / mc(2)155) OX=246196 GN=MSMEG_0639 PE=4 SV=1 | **FtsK-HT unique** |
| A0QTE7 | MSMEG_1813 | 69 | 2 | 2 |  |  |  | Propionyl-CoA carboxylase beta chain OS=Mycolicibacterium smegmatis (strain ATCC 700084 / mc(2)155) OX=246196 GN=MSMEG_1813 PE=3 SV=1 | **FtsK-HT unique** |
| A0QQF9 | MSMEG_0741 | 58 | 2 | 2 |  |  |  | Luciferase-like domain-containing protein OS=Mycolicibacterium smegmatis (strain ATCC 700084 / mc(2)155) OX=246196 GN=MSMEG_0741 PE=4 SV=1 | **FtsK-HT unique** |
| A0R6D6 | MSMEG_6511 | 55 | 1 | 1 |  |  |  | Acyl-CoA dehydrogenase domain protein OS=Mycolicibacterium smegmatis (strain ATCC 700084 / mc(2)155) OX=246196 GN=MSMEG_6511 PE=3 SV=1 | **FtsK-HT unique** |
| A0QP93 | MSMEG_0317 | 52 | 2 | 1 |  |  |  | Integral membrane protein OS=Mycolicibacterium smegmatis (strain ATCC 700084 / mc(2)155) OX=246196 GN=MSMEG_0317 PE=1 SV=1 | **FtsK-HT unique** |
| A0R2Q7 | MSMEG_5199 | 52 | 2 | 2 |  |  |  | Acyl-CoA dehydrogenase OS=Mycolicibacterium smegmatis (strain ATCC 700084 / mc(2)155) OX=246196 GN=MSMEG_5199 PE=3 SV=1 | **FtsK-HT unique** |
| A0QTT7 | MSMEG_1959 MSMEI_1915 | 47 | 2 | 2 |  |  |  | UPF0182 protein MSMEG_1959/MSMEI_1915 OS=Mycolicibacterium smegmatis (strain ATCC 700084 / mc(2)155) OX=246196 GN=MSMEG_1959 PE=1 SV=1 | **FtsK-HT unique** |
| A0QYR7 | MSMEG_3761 | 47 | 2 | 1 |  |  |  | Clp protease subunit OS=Mycolicibacterium smegmatis (strain ATCC 700084 / mc(2)155) OX=246196 GN=MSMEG_3761 PE=4 SV=1 | **FtsK-HT unique** |
| A0R2U8 | fumC MSMEG_5240 | 45 | 2 | 1 |  |  |  | Fumarate hydratase class II OS=Mycolicibacterium smegmatis (strain ATCC 700084 / mc(2)155) OX=246196 GN=fumC PE=1 SV=1 | **FtsK-HT unique** |
| A0R638 | serS MSMEG_6413 MSMEI_6245 | 44 | 1 | 1 |  |  |  | Serine--tRNA ligase OS=Mycolicibacterium smegmatis (strain ATCC 700084 / mc(2)155) OX=246196 GN=serS PE=1 SV=1 | **FtsK-HT unique** |
| A0R0E9 | MSMEG_4362 | 43 | 1 | 1 |  |  |  | Universal stress protein family protein OS=Mycolicibacterium smegmatis (strain ATCC 700084 / mc(2)155) OX=246196 GN=MSMEG_4362 PE=3 SV=1 | **FtsK-HT unique** |
| A0QV12 | MSMEG_2410 | 42 | 1 | 1 |  |  |  | Serine-threonine protein kinase OS=Mycolicibacterium smegmatis (strain ATCC 700084 / mc(2)155) OX=246196 GN=MSMEG_2410 PE=4 SV=1 | **FtsK-HT unique** |
| A0R3L4 | purH MSMEG_5515 | 42 | 1 | 1 |  |  |  | Bifunctional purine biosynthesis protein PurH OS=Mycolicibacterium smegmatis (strain ATCC 700084 / mc(2)155) OX=246196 GN=purH PE=3 SV=1 | **FtsK-HT unique** |
| A0QYF5 | glcB MSMEG_3640 | 41 | 1 | 1 |  |  |  | Malate synthase G OS=Mycolicibacterium smegmatis (strain ATCC 700084 / mc(2)155) OX=246196 GN=glcB PE=3 SV=1 | **FtsK-HT unique** |
| A0QWX9 | tal MSMEG_3102 MSMEI_3024 | 38 | 2 | 1 |  |  |  | Transaldolase OS=Mycolicibacterium smegmatis (strain ATCC 700084 / mc(2)155) OX=246196 GN=tal PE=1 SV=1 | **FtsK-HT unique** |
| A0QNZ3 | MSMEG_0216 | 35 | 1 | 1 |  |  |  | 3-hydroxyacyl-CoA dehydrogenase OS=Mycolicibacterium smegmatis (strain ATCC 700084 / mc(2)155) OX=246196 GN=MSMEG_0216 PE=3 SV=1 | **FtsK-HT unique** |
| A0R628 | glfT2 MSMEG_6403 MSMEI_6235 | 35 | 2 | 1 |  |  |  | Galactofuranosyltransferase GlfT2 OS=Mycolicibacterium smegmatis (strain ATCC 700084 / mc(2)155) OX=246196 GN=glfT2 PE=3 SV=1 | **FtsK-HT unique** |
| A0QWQ4 | alaS MSMEG_3025 MSMEI_2950 | 34 | 1 | 1 |  |  |  | Alanine--tRNA ligase OS=Mycolicibacterium smegmatis (strain ATCC 700084 / mc(2)155) OX=246196 GN=alaS PE=1 SV=1 | **FtsK-HT unique** |
| A0R5K8 | MSMEG_6227 | 34 | 1 | 1 |  |  |  | Transcriptional regulator PadR family protein OS=Mycolicibacterium smegmatis (strain ATCC 700084 / mc(2)155) OX=246196 GN=MSMEG_6227 PE=4 SV=1 | **FtsK-HT unique** |
| A0R0B0 | aceE MSMEG_4323 MSMEI_4223 | 32 | 2 | 1 |  |  |  | Pyruvate dehydrogenase E1 component OS=Mycolicibacterium smegmatis (strain ATCC 700084 / mc(2)155) OX=246196 GN=aceE PE=1 SV=1 | **FtsK-HT unique** |
| A0R2G5 | dapD MSMEG_5104 MSMEI_4976 | 32 | 2 | 1 |  |  |  | 2345-tetrahydropyridine-26-dicarboxylate N-succinyltransferase OS=Mycolicibacterium smegmatis (strain ATCC 700084 / mc(2)155) OX=246196 GN=dapD PE=3 SV=1 | **FtsK-HT unique** |
| A0QQB0 | MSMEG_0690 | 31 | 1 | 1 |  |  |  | Iron-sulfur cluster-binding protein OS=Mycolicibacterium smegmatis (strain ATCC 700084 / mc(2)155) OX=246196 GN=MSMEG_0690 PE=4 SV=1 | **FtsK-HT unique** |
| A0QTE3 | MSMEG_1809 | 31 | 1 | 1 |  |  |  | Sulfurtransferase OS=Mycolicibacterium smegmatis (strain ATCC 700084 / mc(2)155) OX=246196 GN=MSMEG_1809 PE=4 SV=1 | **FtsK-HT unique** |
| A0QTP4 | MSMEG_1916 | 30 | 1 | 1 |  |  |  | Uncharacterized protein OS=Mycolicibacterium smegmatis (strain ATCC 700084 / mc(2)155) OX=246196 GN=MSMEG_1916 PE=1 SV=1 | **FtsK-HT unique** |
| A0QWV9 | whiA MSMEG_3081 MSMEI_3003 | 30 | 1 | 1 |  |  |  | Probable cell division protein WhiA OS=Mycolicibacterium smegmatis (strain ATCC 700084 / mc(2)155) OX=246196 GN=whiA PE=1 SV=3 | **FtsK-HT unique** |
| A0QR00 | gpmA MSMEG_0935 | 29 | 2 | 1 |  |  |  | 23-bisphosphoglycerate-dependent phosphoglycerate mutase OS=Mycolicibacterium smegmatis (strain ATCC 700084 / mc(2)155) OX=246196 GN=gpmA PE=3 SV=1 | **FtsK-HT unique** |
| A0QR91 | MSMEG_1030 MSMEG_2310 | 29 | 1 | 1 |  |  |  | Monooxygenase OS=Mycolicibacterium smegmatis (strain ATCC 700084 / mc(2)155) OX=246196 GN=MSMEG_1030 PE=3 SV=1 | **FtsK-HT unique** |
| A0R042 | MSMEG_4254 | 29 | 1 | 1 |  |  |  | Acyl-CoA synthetase OS=Mycolicibacterium smegmatis (strain ATCC 700084 / mc(2)155) OX=246196 GN=MSMEG_4254 PE=4 SV=1 | **FtsK-HT unique** |
| A0R7G5 | MSMEG_6903 | 28 | 1 | 1 |  |  |  | Transcriptional regulator PadR family protein OS=Mycolicibacterium smegmatis (strain ATCC 700084 / mc(2)155) OX=246196 GN=MSMEG_6903 PE=4 SV=1 | **FtsK-HT unique** |
| A0R066 | ilvE MSMEG_4276 MSMEI_4176 | 25 | 1 | 1 |  |  |  | Branched-chain-amino-acid aminotransferase OS=Mycolicibacterium smegmatis (strain ATCC 700084 / mc(2)155) OX=246196 GN=ilvE PE=1 SV=1 | **FtsK-HT unique** |
| A0R2Y2 | purT MSMEG_5274 MSMEI_5135 | 24 | 1 | 1 |  |  |  | Formate-dependent phosphoribosylglycinamide formyltransferase OS=Mycolicibacterium smegmatis (strain ATCC 700084 / mc(2)155) OX=246196 GN=purT PE=3 SV=1 | **FtsK-HT unique** |
| A0QNN8 | pntA MSMEG_0110 | 23 | 1 | 1 |  |  |  | proton-translocating NAD(P)(+) transhydrogenase OS=Mycolicibacterium smegmatis (strain ATCC 700084 / mc(2)155) OX=246196 GN=pntA PE=3 SV=1 | **FtsK-HT unique** |
| A0QT18 | MSMEG_1680 | 22 | 1 | 1 |  |  |  | Fimbrial assembly protein FimA OS=Mycolicibacterium smegmatis (strain ATCC 700084 / mc(2)155) OX=246196 GN=MSMEG_1680 PE=4 SV=1 | **FtsK-HT unique** |
| A0QWZ9 | sufB MSMEG_3122 | 22 | 1 | 1 |  |  |  | FeS assembly protein SufB OS=Mycolicibacterium smegmatis (strain ATCC 700084 / mc(2)155) OX=246196 GN=sufB PE=3 SV=1 | **FtsK-HT unique** |
| A0R090 | MSMEG_4301 | 21 | 1 | 1 |  |  |  | Acyl-CoA synthase OS=Mycolicibacterium smegmatis (strain ATCC 700084 / mc(2)155) OX=246196 GN=MSMEG_4301 PE=4 SV=1 | **FtsK-HT unique** |
| A0QYH8 | MSMEG_3663 | 20 | 1 | 1 |  |  |  | Oxidoreductase OS=Mycolicibacterium smegmatis (strain ATCC 700084 / mc(2)155) OX=246196 GN=MSMEG_3663 PE=4 SV=1 | **FtsK-HT unique** |
| A0R152 | rne MSMEG_4626 MSMEI_4509 | 20 | 1 | 1 |  |  |  | Ribonuclease E OS=Mycolicibacterium smegmatis (strain ATCC 700084 / mc(2)155) OX=246196 GN=rne PE=1 SV=1 | **FtsK-HT unique** |
| A0QPV4 | MSMEG_0531 | 19 | 1 | 1 |  |  |  | Acyl-CoA dehydrogenase OS=Mycolicibacterium smegmatis (strain ATCC 700084 / mc(2)155) OX=246196 GN=MSMEG_0531 PE=3 SV=1 | **FtsK-HT unique** |
| A0QS44 | nusG MSMEG_1345 | 19 | 1 | 1 |  |  |  | Transcription termination/antitermination protein NusG OS=Mycolicibacterium smegmatis (strain ATCC 700084 / mc(2)155) OX=246196 GN=nusG PE=3 SV=1 | **FtsK-HT unique** |
| A0QWW4 | tpiA MSMEG_3086 | 19 | 2 | 1 |  |  |  | Triosephosphate isomerase OS=Mycolicibacterium smegmatis (strain ATCC 700084 / mc(2)155) OX=246196 GN=tpiA PE=3 SV=1 | **FtsK-HT unique** |
| A0QX21 | MSMEG_3144 | 19 | 1 | 1 |  |  |  | Membrane protein OS=Mycolicibacterium smegmatis (strain ATCC 700084 / mc(2)155) OX=246196 GN=MSMEG_3144 PE=4 SV=1 | **FtsK-HT unique** |
| A0QZ54 | mpa MSMEG_3902 MSMEI_3813 | 18 | 1 | 1 |  |  |  | Proteasome-associated ATPase OS=Mycolicibacterium smegmatis (strain ATCC 700084 / mc(2)155) OX=246196 GN=mpa PE=1 SV=1 | **FtsK-HT unique** |
| A0QNN7 | pntB MSMEG_0109 | 17 | 1 | 1 |  |  |  | NAD(P) transhydrogenase subunit beta OS=Mycolicibacterium smegmatis (strain ATCC 700084 / mc(2)155) OX=246196 GN=pntB PE=3 SV=1 | **FtsK-HT unique** |
| A0QR73 | MSMEG_1010 MSMEG_2290 | 17 | 1 | 1 |  |  |  | Transcriptional regulator TetR family protein OS=Mycolicibacterium smegmatis (strain ATCC 700084 / mc(2)155) OX=246196 GN=MSMEG_1010 PE=4 SV=1 | **FtsK-HT unique** |
| A0QX85 | priA hisA MSMEG_3209 | 17 | 1 | 1 |  |  |  | Phosphoribosyl isomerase A OS=Mycolicibacterium smegmatis (strain ATCC 700084 / mc(2)155) OX=246196 GN=priA PE=3 SV=1 | **FtsK-HT unique** |
| A0R6E3 | MSMEG_6518 MSMEI_6344 | 16 | 1 | 1 |  |  |  | Uncharacterized protein MSMEG_6518/MSMEI_6344 OS=Mycolicibacterium smegmatis (strain ATCC 700084 / mc(2)155) OX=246196 GN=MSMEG_6518 PE=1 SV=1 | **FtsK-HT unique** |
| A0QPE8 | MSMEG_0373 | 15 | 1 | 1 |  |  |  | 3-ketoacyl-CoA thiolase OS=Mycolicibacterium smegmatis (strain ATCC 700084 / mc(2)155) OX=246196 GN=MSMEG_0373 PE=3 SV=1 | **FtsK-HT unique** |
| A0QP15 | MSMEG_0238 |  |  |  | 46 | 2 | 2 | O-acetylhomoserine/O-acetylserine sulfhydrylase OS=Mycolicibacterium smegmatis (strain ATCC 700084 / mc(2)155) OX=246196 GN=MSMEG_0238 PE=4 SV=1 | **sFtsK-HT unique** |
| A0QP20 | MSMEG_0243 |  |  |  | 35 | 2 | 1 | Haemophore haem-binding domain-containing protein OS=Mycolicibacterium smegmatis (strain ATCC 700084 / mc(2)155) OX=246196 GN=MSMEG_0243 PE=4 SV=1 | **sFtsK-HT unique** |
| A0QPH5 | MSMEG_0400 |  |  |  | 26 | 1 | 1 | Peptide synthetase OS=Mycolicibacterium smegmatis (strain ATCC 700084 / mc(2)155) OX=246196 GN=MSMEG_0400 PE=4 SV=1 | **sFtsK-HT unique** |
| A0QPT8 | MSMEG_0514 |  |  |  | 20 | 1 | 1 | Alpha-galactosidase OS=Mycolicibacterium smegmatis (strain ATCC 700084 / mc(2)155) OX=246196 GN=MSMEG_0514 PE=3 SV=1 | **sFtsK-HT unique** |
| A0QQF4 | ttfA MSMEG_0736 MSMEI_0720 |  |  |  | 42 | 2 | 2 | Trehalose monomycolate transport factor A OS=Mycolicibacterium smegmatis (strain ATCC 700084 / mc(2)155) OX=246196 GN=ttfA PE=1 SV=1 | **sFtsK-HT unique** |
| A0QQN1 | MSMEG_0814 |  |  |  | 18 | 1 | 1 | Membrane protein OS=Mycolicibacterium smegmatis (strain ATCC 700084 / mc(2)155) OX=246196 GN=MSMEG_0814 PE=4 SV=1 | **sFtsK-HT unique** |
| A0QR09 | MSMEG_0944 |  |  |  | 22 | 1 | 1 | DNA binding domain excisionase family protein OS=Mycolicibacterium smegmatis (strain ATCC 700084 / mc(2)155) OX=246196 GN=MSMEG_0944 PE=4 SV=1 | **sFtsK-HT unique** |
| A0QRB4 | MSMEG_1055 MSMEG_2335 |  |  |  | 41 | 1 | 1 | Hexapeptide transferase family protein OS=Mycolicibacterium smegmatis (strain ATCC 700084 / mc(2)155) OX=246196 GN=MSMEG_1055 PE=4 SV=1 | **sFtsK-HT unique** |
| A0QRY7 | MSMEG_1285 |  |  |  | 35 | 1 | 1 | Tetratricopeptide repeat family protein OS=Mycolicibacterium smegmatis (strain ATCC 700084 / mc(2)155) OX=246196 GN=MSMEG_1285 PE=4 SV=1 | **sFtsK-HT unique** |
| A0QSL0 | MSMEG_1516 |  |  |  | 20 | 1 | 1 | Thioredoxin reductase OS=Mycolicibacterium smegmatis (strain ATCC 700084 / mc(2)155) OX=246196 GN=MSMEG_1516 PE=4 SV=1 | **sFtsK-HT unique** |
| A0QSN7 | MSMEG_1543 |  |  |  | 55 | 2 | 1 | Eptc-inducible aldehyde dehydrogenase OS=Mycolicibacterium smegmatis (strain ATCC 700084 / mc(2)155) OX=246196 GN=MSMEG_1543 PE=3 SV=1 | **sFtsK-HT unique** |
| A0QSU5 | MSMEG_1604 |  |  |  | 21 | 1 | 1 | FAD dependent oxidoreductase OS=Mycolicibacterium smegmatis (strain ATCC 700084 / mc(2)155) OX=246196 GN=MSMEG_1604 PE=4 SV=1 | **sFtsK-HT unique** |
| A0QSY7 | MSMEG_1648 |  |  |  | 16 | 1 | 1 | Transcriptional regulator OS=Mycolicibacterium smegmatis (strain ATCC 700084 / mc(2)155) OX=246196 GN=MSMEG_1648 PE=4 SV=1 | **sFtsK-HT unique** |
| A0QTD6 | MSMEG_1802 |  |  |  | 18 | 1 | 1 | ChaB protein OS=Mycolicibacterium smegmatis (strain ATCC 700084 / mc(2)155) OX=246196 GN=MSMEG_1802 PE=4 SV=1 | **sFtsK-HT unique** |
| A0QTE6 | MSMEG_1812 |  |  |  | 21 | 1 | 1 | Acetyl-/propionyl-coenzyme A carboxylase AccE5 OS=Mycolicibacterium smegmatis (strain ATCC 700084 / mc(2)155) OX=246196 GN=MSMEG_1812 PE=4 SV=1 | **sFtsK-HT unique** |
| A0QTF4 | MSMEG_1821 |  |  |  | 59 | 2 | 1 | Acyl-CoA dehydrogenase OS=Mycolicibacterium smegmatis (strain ATCC 700084 / mc(2)155) OX=246196 GN=MSMEG_1821 PE=3 SV=1 | **sFtsK-HT unique** |
| A0QTP8 | MSMEG_1920 |  |  |  | 42 | 1 | 1 | Diacylglycerol kinase catalytic region OS=Mycolicibacterium smegmatis (strain ATCC 700084 / mc(2)155) OX=246196 GN=MSMEG_1920 PE=4 SV=1 | **sFtsK-HT unique** |
| A0QUY7 | gltX MSMEG_2383 MSMEI_2323 |  |  |  | 49 | 2 | 2 | Glutamate--tRNA ligase OS=Mycolicibacterium smegmatis (strain ATCC 700084 / mc(2)155) OX=246196 GN=gltX PE=1 SV=1 | **sFtsK-HT unique** |
| A0QVP6 | MSMEG_2647 |  |  |  | 44 | 2 | 2 | Metallophosphoesterase OS=Mycolicibacterium smegmatis (strain ATCC 700084 / mc(2)155) OX=246196 GN=MSMEG_2647 PE=4 SV=1 | **sFtsK-HT unique** |
| A0QWU7 | mfs MSMEG_3069 MSMEI_2992 |  |  |  | 27 | 1 | 1 | Probable triacylglyceride transporter MSMEG_3069/MSMEI_2992 OS=Mycolicibacterium smegmatis (strain ATCC 700084 / mc(2)155) OX=246196 GN=MSMEG_3069 PE=3 SV=1 | **sFtsK-HT unique** |
| A0QX80 | MSMEG_3204 |  |  |  | 80 | 4 | 1 | Nitroreductase OS=Mycolicibacterium smegmatis (strain ATCC 700084 / mc(2)155) OX=246196 GN=MSMEG_3204 PE=4 SV=1 | **sFtsK-HT unique** |
| A0QYD6 | ndh MSMEG_3621 |  |  |  | 50 | 3 | 2 | NADH:ubiquinone reductase (non-electrogenic) OS=Mycolicibacterium smegmatis (strain ATCC 700084 / mc(2)155) OX=246196 GN=ndh PE=3 SV=1 | **sFtsK-HT unique** |
| A0QYG2 | garA MSMEG_3647 MSMEI_3561 |  |  |  | 39 | 1 | 1 | Glycogen accumulation regulator GarA OS=Mycolicibacterium smegmatis (strain ATCC 700084 / mc(2)155) OX=246196 GN=garA PE=1 SV=2 | **sFtsK-HT unique** |
| A0QYK4 | MSMEG_3689 |  |  |  | 33 | 1 | 1 | Sodium:solute symporter OS=Mycolicibacterium smegmatis (strain ATCC 700084 / mc(2)155) OX=246196 GN=MSMEG_3689 PE=3 SV=1 | **sFtsK-HT unique** |
| A0QZX1 | hisG MSMEG_4180 |  |  |  | 28 | 1 | 1 | ATP phosphoribosyltransferase OS=Mycolicibacterium smegmatis (strain ATCC 700084 / mc(2)155) OX=246196 GN=hisG PE=3 SV=1 | **sFtsK-HT unique** |
| A0R0B5 | MSMEG_4328 |  |  |  | 62 | 3 | 2 | 3-oxoacyl-[acyl-carrier-protein] synthase 2 OS=Mycolicibacterium smegmatis (strain ATCC 700084 / mc(2)155) OX=246196 GN=MSMEG_4328 PE=3 SV=1 | **sFtsK-HT unique** |
| A0R0W7 | MSMEG_4533 |  |  |  | 27 | 2 | 1 | Sulfate-binding protein OS=Mycolicibacterium smegmatis (strain ATCC 700084 / mc(2)155) OX=246196 GN=MSMEG_4533 PE=3 SV=1 | **sFtsK-HT unique** |
| A0R103 | MSMEG_4572 |  |  |  | 41 | 2 | 2 | DNA polymerase III delta subunit OS=Mycolicibacterium smegmatis (strain ATCC 700084 / mc(2)155) OX=246196 GN=MSMEG_4572 PE=4 SV=1 | **sFtsK-HT unique** |
| A0R1Y8 | mce MSMEG_4921 |  |  |  | 21 | 1 | 1 | Methylmalonyl-CoA epimerase OS=Mycolicibacterium smegmatis (strain ATCC 700084 / mc(2)155) OX=246196 GN=mce PE=3 SV=1 | **sFtsK-HT unique** |
| A0R2E8 | MSMEG_5086 |  |  |  | 35 | 1 | 1 | Very-long-chain acyl-CoA synthetase OS=Mycolicibacterium smegmatis (strain ATCC 700084 / mc(2)155) OX=246196 GN=MSMEG_5086 PE=3 SV=1 | **sFtsK-HT unique** |
| A0R3D3 | pth MSMEG_5432 MSMEI_5283 |  |  |  | 21 | 1 | 1 | Peptidyl-tRNA hydrolase OS=Mycolicibacterium smegmatis (strain ATCC 700084 / mc(2)155) OX=246196 GN=pth PE=1 SV=1 | **sFtsK-HT unique** |
| A0R3M5 | MSMEG_5526 |  |  |  | 15 | 1 | 1 | Peptidoglycan-binding LysM OS=Mycolicibacterium smegmatis (strain ATCC 700084 / mc(2)155) OX=246196 GN=MSMEG_5526 PE=4 SV=1 | **sFtsK-HT unique** |
| A0R3N3 | pcrA MSMEG_5534 |  |  |  | 15 | 1 | 1 | ATP-dependent DNA helicase OS=Mycolicibacterium smegmatis (strain ATCC 700084 / mc(2)155) OX=246196 GN=pcrA PE=3 SV=1 | **sFtsK-HT unique** |
| A0R4B1 | MSMEG_5770 |  |  |  | 21 | 1 | 1 | Uncharacterized protein OS=Mycolicibacterium smegmatis (strain ATCC 700084 / mc(2)155) OX=246196 GN=MSMEG_5770 PE=4 SV=1 | **sFtsK-HT unique** |
| A0R574 | clpC1 MSMEG_6091 MSMEI_5933 |  |  |  | 54 | 1 | 1 | ATP-dependent Clp protease ATP-binding subunit ClpC1 OS=Mycolicibacterium smegmatis (strain ATCC 700084 / mc(2)155) OX=246196 GN=clpC1 PE=3 SV=1 | **sFtsK-HT unique** |
| A0R5M2 | MSMEG_6241 |  |  |  | 44 | 1 | 1 | ATPase associated with various cellular activities AAA-5 OS=Mycolicibacterium smegmatis (strain ATCC 700084 / mc(2)155) OX=246196 GN=MSMEG_6241 PE=4 SV=1 | **sFtsK-HT unique** |
| A0R5R5 | MSMEG_6284 |  |  |  | 55 | 1 | 1 | Cyclopropane-fatty-acyl-phospholipid synthase OS=Mycolicibacterium smegmatis (strain ATCC 700084 / mc(2)155) OX=246196 GN=MSMEG_6284 PE=3 SV=1 | **sFtsK-HT unique** |
| A0R617 | MSMEG_6392 |  |  |  | 45 | 2 | 2 | Polyketide synthase OS=Mycolicibacterium smegmatis (strain ATCC 700084 / mc(2)155) OX=246196 GN=MSMEG_6392 PE=4 SV=1 | **sFtsK-HT unique** |
| Q9AFI5 | KRT84 KRTHB4 |  |  |  | 30 | 1 | 1 | Single-stranded DNA-binding protein OS=Mycolicibacterium smegmatis (strain ATCC 700084 / mc(2)155) OX=246196 GN=ssb PE=1 SV=1 | **sFtsK-HT unique** |
| Q9NSB2 | glgE MSMEG_4916 MSMEI_4789 |  |  |  | 20 | 1 | 1 | Keratin type II cuticular Hb4 OS=Homo sapiens OX=9606 GN=KRT84 PE=1 SV=2 | **sFtsK-HT unique** |
| A0QSD4 | rplB MSMEG_1439 MSMEI_1403 | 15858 | 69 | 4 | 18401 | 64 | 4 | Large ribosomal subunit protein uL2 OS=Mycolicibacterium smegmatis (strain ATCC 700084 / mc(2)155) OX=246196 GN=rplB PE=1 SV=1 | **Shared** |
| A0QS66 | rpoC MSMEG_1368 MSMEI_1329 | 9764 | 195 | 4 | 15443 | 236 | 4 | DNA-directed RNA polymerase subunit beta~ OS=Mycolicibacterium smegmatis (strain ATCC 700084 / mc(2)155) OX=246196 GN=rpoC PE=1 SV=1 | **Shared** |
| A0QVT6 | MSMEG_2690 | 4250 | 97 | 4 | 5457 | 98 | 4 | DNA translocase FtsK OS=Mycolicibacterium smegmatis (strain ATCC 700084 / mc(2)155) OX=246196 GN=MSMEG_2690 PE=3 SV=1 | **Shared** |
| A0QYU6 | rplT MSMEG_3791 MSMEI_3703 | 4184 | 38 | 4 | 2834 | 30 | 4 | Large ribosomal subunit protein bL20 OS=Mycolicibacterium smegmatis (strain ATCC 700084 / mc(2)155) OX=246196 GN=rplT PE=1 SV=1 | **Shared** |
| A0QSP9 | rpsI MSMEG_1557 MSMEI_1520 | 4075 | 49 | 4 | 5315 | 47 | 4 | Small ribosomal subunit protein uS9 OS=Mycolicibacterium smegmatis (strain ATCC 700084 / mc(2)155) OX=246196 GN=rpsI PE=1 SV=1 | **Shared** |
| A0QSG8 | rplO MSMEG_1474 | 4008 | 33 | 4 | 3153 | 30 | 4 | Large ribosomal subunit protein uL15 OS=Mycolicibacterium smegmatis (strain ATCC 700084 / mc(2)155) OX=246196 GN=rplO PE=1 SV=1 | **Shared** |
| A0R069 | pepA MSMEG_4281 | 3093 | 50 | 4 | 1302 | 31 | 4 | Probable cytosol aminopeptidase OS=Mycolicibacterium smegmatis (strain ATCC 700084 / mc(2)155) OX=246196 GN=pepA PE=3 SV=1 | **Shared** |
| A0QSD2 | rplD MSMEG_1437 MSMEI_1401 | 3090 | 41 | 4 | 2334 | 36 | 4 | Large ribosomal subunit protein uL4 OS=Mycolicibacterium smegmatis (strain ATCC 700084 / mc(2)155) OX=246196 GN=rplD PE=1 SV=1 | **Shared** |
| A0QSL7 | rpsD MSMEG_1523 MSMEI_1487 | 2832 | 52 | 4 | 3626 | 56 | 4 | Small ribosomal subunit protein uS4 OS=Mycolicibacterium smegmatis (strain ATCC 700084 / mc(2)155) OX=246196 GN=rpsD PE=1 SV=1 | **Shared** |
| A0R151 | rplU MSMEG_4625 MSMEI_4508 | 2512 | 35 | 4 | 1254 | 28 | 4 | Large ribosomal subunit protein bL21 OS=Mycolicibacterium smegmatis (strain ATCC 700084 / mc(2)155) OX=246196 GN=rplU PE=1 SV=1 | **Shared** |
| A0R076 | MSMEG_4287 | 2456 | 46 | 4 | 2017 | 35 | 4 | Integral membrane protein OS=Mycolicibacterium smegmatis (strain ATCC 700084 / mc(2)155) OX=246196 GN=MSMEG_4287 PE=4 SV=1 | **Shared** |
| P0001 |  | 2247 | 21 | 4 | 2385 | 21 | 4 | HaloTag sequence ZMM_KMI OS=recomb OX=0 GN=a PE=0 SV=0 | **Shared** |
| A0R102 | rpsT MSMEG_4571 MSMEI_4459 | 2193 | 25 | 4 | 2081 | 24 | 4 | Small ribosomal subunit protein bS20 OS=Mycolicibacterium smegmatis (strain ATCC 700084 / mc(2)155) OX=246196 GN=rpsT PE=1 SV=1 | **Shared** |
| A0QSP8 | rplM MSMEG_1556 MSMEI_1519 | 2095 | 33 | 4 | 2379 | 27 | 4 | Large ribosomal subunit protein uL13 OS=Mycolicibacterium smegmatis (strain ATCC 700084 / mc(2)155) OX=246196 GN=rplM PE=1 SV=1 | **Shared** |
| A0R659 | MSMEG_6434 | 2081 | 31 | 4 | 2612 | 23 | 4 | Uncharacterized protein OS=Mycolicibacterium smegmatis (strain ATCC 700084 / mc(2)155) OX=246196 GN=MSMEG_6434 PE=4 SV=1 | **Shared** |
| A0QRB8 | MSMEG_1060 | 1985 | 21 | 4 | 908 | 15 | 4 | Lsr2 protein OS=Mycolicibacterium smegmatis (strain ATCC 700084 / mc(2)155) OX=246196 GN=MSMEG_1060 PE=4 SV=1 | **Shared** |
| A0R729 | glpK MSMEG_6759 | 1906 | 46 | 4 | 357 | 14 | 4 | Glycerol kinase OS=Mycolicibacterium smegmatis (strain ATCC 700084 / mc(2)155) OX=246196 GN=glpK PE=3 SV=1 | **Shared** |
| A0QSL6 | rpsK MSMEG_1522 MSMEI_1486 | 1870 | 18 | 4 | 1532 | 19 | 4 | Small ribosomal subunit protein uS11 OS=Mycolicibacterium smegmatis (strain ATCC 700084 / mc(2)155) OX=246196 GN=rpsK PE=1 SV=1 | **Shared** |
| A0QSL5 | rpsM MSMEG_1521 MSMEI_1485 | 1685 | 36 | 4 | 1719 | 33 | 4 | Small ribosomal subunit protein uS13 OS=Mycolicibacterium smegmatis (strain ATCC 700084 / mc(2)155) OX=246196 GN=rpsM PE=1 SV=1 | **Shared** |
| Q9ZHC5 |  | 1607 | 27 | 4 | 1804 | 23 | 4 | DNA-binding protein HupB OS=Mycolicibacterium smegmatis (strain ATCC 700084 / mc(2)155) OX=246196 GN=hup PE=1 SV=1 | **Shared** |
| A0R2A4 | deaD csdA MSMEG_5042 | 1542 | 42 | 3 | 3532 | 61 | 3 | ATP-dependent RNA helicase DeaD OS=Mycolicibacterium smegmatis (strain ATCC 700084 / mc(2)155) OX=246196 GN=deaD PE=3 SV=1 | **Shared** |
| A0QQU5 | groEL2 groL2 MSMEG_0880 MSMEI_0859 | 1528 | 46 | 4 | 1129 | 34 | 4 | Chaperonin GroEL 2 OS=Mycolicibacterium smegmatis (strain ATCC 700084 / mc(2)155) OX=246196 GN=groEL2 PE=1 SV=1 | **Shared** |
| A0QSD5 | rpsS MSMEG_1440 MSMEI_1404 | 1523 | 17 | 4 | 2247 | 18 | 4 | Small ribosomal subunit protein uS19 OS=Mycolicibacterium smegmatis (strain ATCC 700084 / mc(2)155) OX=246196 GN=rpsS PE=1 SV=1 | **Shared** |
| A0QS98 | tuf MSMEG_1401 MSMEI_1363 | 1512 | 33 | 4 | 981 | 25 | 4 | Elongation factor Tu OS=Mycolicibacterium smegmatis (strain ATCC 700084 / mc(2)155) OX=246196 GN=tuf PE=1 SV=1 | **Shared** |
| A0R091 | MSMEG_4302 | 1501 | 38 | 4 | 2315 | 40 | 4 | Adenylate cyclase putative OS=Mycolicibacterium smegmatis (strain ATCC 700084 / mc(2)155) OX=246196 GN=MSMEG_4302 PE=4 SV=1 | **Shared** |
| A0QS46 | rplA MSMEG_1347 MSMEI_1309 | 1460 | 42 | 4 | 1322 | 38 | 4 | Large ribosomal subunit protein uL1 OS=Mycolicibacterium smegmatis (strain ATCC 700084 / mc(2)155) OX=246196 GN=rplA PE=1 SV=1 | **Shared** |
| A0QVM7 | infB MSMEG_2628 | 1367 | 28 | 4 | 801 | 23 | 4 | Translation initiation factor IF-2 OS=Mycolicibacterium smegmatis (strain ATCC 700084 / mc(2)155) OX=246196 GN=infB PE=3 SV=1 | **Shared** |
| A0QS97 | rpsG MSMEG_1399 MSMEI_1361 | 1354 | 22 | 4 | 1166 | 19 | 4 | Small ribosomal subunit protein uS7 OS=Mycolicibacterium smegmatis (strain ATCC 700084 / mc(2)155) OX=246196 GN=rpsG PE=1 SV=1 | **Shared** |
| A0QSG4 | rplF MSMEG_1470 MSMEI_1434 | 1274 | 37 | 4 | 2159 | 32 | 4 | Large ribosomal subunit protein uL6 OS=Mycolicibacterium smegmatis (strain ATCC 700084 / mc(2)155) OX=246196 GN=rplF PE=1 SV=1 | **Shared** |
| A0R7F7 | rpsR2 rpsR1 MSMEG_6895 MSMEI_6711 | 1264 | 29 | 4 | 1208 | 24 | 4 | Small ribosomal subunit protein bS18B OS=Mycolicibacterium smegmatis (strain ATCC 700084 / mc(2)155) OX=246196 GN=rpsR2 PE=1 SV=1 | **Shared** |
| A0R5M3 | MSMEG_6242 | 1202 | 30 | 4 | 1645 | 24 | 3 | Alcohol dehydrogenase iron-containing OS=Mycolicibacterium smegmatis (strain ATCC 700084 / mc(2)155) OX=246196 GN=MSMEG_6242 PE=3 SV=1 | **Shared** |
| A0QSD1 | rplC MSMEG_1436 MSMEI_1400 | 1190 | 22 | 4 | 1159 | 27 | 4 | Large ribosomal subunit protein uL3 OS=Mycolicibacterium smegmatis (strain ATCC 700084 / mc(2)155) OX=246196 GN=rplC PE=1 SV=1 | **Shared** |
| A0QNG7 | MSMEG_0035 | 1173 | 28 | 4 | 1343 | 29 | 4 | FHA domain protein OS=Mycolicibacterium smegmatis (strain ATCC 700084 / mc(2)155) OX=246196 GN=MSMEG_0035 PE=4 SV=1 | **Shared** |
| A0QV42 | rplS MSMEG_2440 MSMEI_2379 | 1134 | 16 | 4 | 1496 | 14 | 3 | Large ribosomal subunit protein bL19 OS=Mycolicibacterium smegmatis (strain ATCC 700084 / mc(2)155) OX=246196 GN=rplS PE=1 SV=1 | **Shared** |
| A0QSD7 | rpsC MSMEG_1442 MSMEI_1406 | 1120 | 34 | 4 | 1644 | 35 | 4 | Small ribosomal subunit protein uS3 OS=Mycolicibacterium smegmatis (strain ATCC 700084 / mc(2)155) OX=246196 GN=rpsC PE=1 SV=1 | **Shared** |
| A0QSD3 | rplW MSMEG_1438 MSMEI_1402 | 1113 | 24 | 4 | 1279 | 24 | 4 | Large ribosomal subunit protein uL23 OS=Mycolicibacterium smegmatis (strain ATCC 700084 / mc(2)155) OX=246196 GN=rplW PE=1 SV=1 | **Shared** |
| A0QV32 | ffh MSMEG_2430 | 1080 | 30 | 4 | 1553 | 36 | 3 | Signal recognition particle protein ffh OS=Mycolicibacterium smegmatis (strain ATCC 700084 / mc(2)155) OX=246196 GN=ffh PE=3 SV=1 | **Shared** |
| A0QTE1 | MSMEG_1807 | 1062 | 39 | 4 | 989 | 28 | 4 | biotin carboxylase OS=Mycolicibacterium smegmatis (strain ATCC 700084 / mc(2)155) OX=246196 GN=MSMEG_1807 PE=4 SV=1 | **Shared** |
| A0QYF6 | MSMEG_3641 | 951 | 22 | 3 | 380 | 11 | 4 | VWFA domain-containing protein OS=Mycolicibacterium smegmatis (strain ATCC 700084 / mc(2)155) OX=246196 GN=MSMEG_3641 PE=4 SV=1 | **Shared** |
| A0QYI8 | malQ MSMEG_3673 | 931 | 32 | 3 | 1340 | 44 | 4 | 4-alpha-glucanotransferase OS=Mycolicibacterium smegmatis (strain ATCC 700084 / mc(2)155) OX=246196 GN=malQ PE=3 SV=1 | **Shared** |
| A0R3I9 | rpmF MSMEG_5489 MSMEI_5337 | 869 | 8 | 4 | 575 | 8 | 4 | Large ribosomal subunit protein bL32 OS=Mycolicibacterium smegmatis (strain ATCC 700084 / mc(2)155) OX=246196 GN=rpmF PE=1 SV=1 | **Shared** |
| A0QSD0 | rpsJ MSMEG_1435 MSMEI_1399 | 832 | 28 | 4 | 770 | 23 | 4 | Small ribosomal subunit protein uS10 OS=Mycolicibacterium smegmatis (strain ATCC 700084 / mc(2)155) OX=246196 GN=rpsJ PE=1 SV=1 | **Shared** |
| A0QSG0 | rplX MSMEG_1466 MSMEI_1430 | 828 | 18 | 4 | 742 | 15 | 4 | Large ribosomal subunit protein uL24 OS=Mycolicibacterium smegmatis (strain ATCC 700084 / mc(2)155) OX=246196 GN=rplX PE=1 SV=1 | **Shared** |
| A0QWH1 | MSMEG_2940 MSMEI_2866 | 806 | 20 | 4 | 558 | 15 | 4 | Probable transcriptional regulatory protein MSMEG_2940/MSMEI_2866 OS=Mycolicibacterium smegmatis (strain ATCC 700084 / mc(2)155) OX=246196 GN=MSMEG_2940 PE=1 SV=1 | **Shared** |
| A0QSL8 | rpoA MSMEG_1524 MSMEI_1488 | 802 | 23 | 4 | 808 | 17 | 4 | DNA-directed RNA polymerase subunit alpha OS=Mycolicibacterium smegmatis (strain ATCC 700084 / mc(2)155) OX=246196 GN=rpoA PE=1 SV=1 | **Shared** |
| A0QYU8 | infC MSMEG_3793 | 786 | 27 | 4 | 858 | 22 | 4 | Translation initiation factor IF-3 OS=Mycolicibacterium smegmatis (strain ATCC 700084 / mc(2)155) OX=246196 GN=infC PE=3 SV=1 | **Shared** |
| A0R218 | rho MSMEG_4954 | 776 | 27 | 4 | 983 | 26 | 4 | Transcription termination factor Rho OS=Mycolicibacterium smegmatis (strain ATCC 700084 / mc(2)155) OX=246196 GN=rho PE=3 SV=1 | **Shared** |
| A0QSE0 | rpsQ MSMEG_1445 MSMEI_1409 | 775 | 24 | 4 | 835 | 20 | 4 | Small ribosomal subunit protein uS17 OS=Mycolicibacterium smegmatis (strain ATCC 700084 / mc(2)155) OX=246196 GN=rpsQ PE=1 SV=1 | **Shared** |
| A0R1H7 | MSMEG_4757 | 708 | 29 | 4 | 557 | 27 | 4 | Fatty acid synthase OS=Mycolicibacterium smegmatis (strain ATCC 700084 / mc(2)155) OX=246196 GN=MSMEG_4757 PE=1 SV=1 | **Shared** |
| P71533 | mabA fabG MSMEG_3150 MSMEI_3069 | 706 | 31 | 4 | 1389 | 34 | 2 | Protein translocase subunit SecA 1 OS=Mycolicibacterium smegmatis (strain ATCC 700084 / mc(2)155) OX=246196 GN=secA1 PE=1 SV=2 | **Shared** |
| A0QW71 | MSMEG_2839 | 657 | 19 | 4 | 370 | 9 | 4 | Transcriptional accessory protein OS=Mycolicibacterium smegmatis (strain ATCC 700084 / mc(2)155) OX=246196 GN=MSMEG_2839 PE=4 SV=1 | **Shared** |
| A0R1C3 | ettA MSMEG_4700 | 620 | 22 | 4 | 691 | 20 | 3 | Energy-dependent translational throttle protein EttA OS=Mycolicibacterium smegmatis (strain ATCC 700084 / mc(2)155) OX=246196 GN=ettA PE=3 SV=1 | **Shared** |
| A0QVB8 | rpsB MSMEG_2519 MSMEI_2460 | 616 | 16 | 4 | 712 | 18 | 4 | Small ribosomal subunit protein uS2 OS=Mycolicibacterium smegmatis (strain ATCC 700084 / mc(2)155) OX=246196 GN=rpsB PE=1 SV=2 | **Shared** |
| A0QSG5 | rplR MSMEG_1471 | 611 | 13 | 4 | 522 | 11 | 4 | Large ribosomal subunit protein uL18 OS=Mycolicibacterium smegmatis (strain ATCC 700084 / mc(2)155) OX=246196 GN=rplR PE=1 SV=1 | **Shared** |
| P13645 | gyrA MSMEG_0006 MSMEI_0008 | 609 | 18 | 3 | 592 | 18 | 3 | Keratin type I cytoskeletal 10 OS=Homo sapiens OX=9606 GN=KRT10 PE=1 SV=6 | **Shared** |
| P60281 | secA1 MSMEG_1881 MSMEI_1840 | 605 | 27 | 4 | 503 | 19 | 4 | DNA-directed RNA polymerase subunit beta OS=Mycolicibacterium smegmatis (strain ATCC 700084 / mc(2)155) OX=246196 GN=rpoB PE=1 SV=1 | **Shared** |
| A0QQK3 | pknG MSMEG_0786 MSMEI_0770 | 586 | 20 | 3 | 445 | 15 | 4 | Serine/threonine-protein kinase PknG OS=Mycolicibacterium smegmatis (strain ATCC 700084 / mc(2)155) OX=246196 GN=pknG PE=1 SV=1 | **Shared** |
| A0QSG2 | rpsZ rpsN MSMEG_1468 MSMEI_1432 | 550 | 4 | 4 | 619 | 4 | 4 | Small ribosomal subunit protein uS14B OS=Mycolicibacterium smegmatis (strain ATCC 700084 / mc(2)155) OX=246196 GN=rpsZ PE=1 SV=1 | **Shared** |
| A0QYY6 | rpsA MSMEG_3833 MSMEI_3743 LJ00_19040 | 530 | 22 | 4 | 283 | 10 | 3 | Small ribosomal subunit protein bS1 OS=Mycolicibacterium smegmatis (strain ATCC 700084 / mc(2)155) OX=246196 GN=rpsA PE=1 SV=1 | **Shared** |
| A0QSS4 | groEL1 groL1 MSMEG_1583 MSMEI_1545 | 517 | 17 | 4 | 243 | 7 | 3 | Chaperonin GroEL 1 OS=Mycolicibacterium smegmatis (strain ATCC 700084 / mc(2)155) OX=246196 GN=groEL1 PE=1 SV=1 | **Shared** |
| A0R062 | MSMEG_4273 | 513 | 17 | 4 | 664 | 17 | 4 | Integral membrane protein OS=Mycolicibacterium smegmatis (strain ATCC 700084 / mc(2)155) OX=246196 GN=MSMEG_4273 PE=4 SV=1 | **Shared** |
| A0QV37 | rpsP MSMEG_2435 MSMEI_2374 | 499 | 17 | 4 | 627 | 15 | 4 | Small ribosomal subunit protein bS16 OS=Mycolicibacterium smegmatis (strain ATCC 700084 / mc(2)155) OX=246196 GN=rpsP PE=1 SV=1 | **Shared** |
| A0R150 | rpmA MSMEG_4624 MSMEI_4507 | 496 | 14 | 4 | 465 | 17 | 4 | Large ribosomal subunit protein bL27 OS=Mycolicibacterium smegmatis (strain ATCC 700084 / mc(2)155) OX=246196 GN=rpmA PE=1 SV=1 | **Shared** |
| A0QXA3 | pyk MSMEG_3227 | 492 | 13 | 4 | 300 | 7 | 3 | Pyruvate kinase OS=Mycolicibacterium smegmatis (strain ATCC 700084 / mc(2)155) OX=246196 GN=pyk PE=3 SV=1 | **Shared** |
| A0QSG1 | rplE MSMEG_1467 MSMEI_1431 | 486 | 13 | 4 | 310 | 8 | 3 | Large ribosomal subunit protein uL5 OS=Mycolicibacterium smegmatis (strain ATCC 700084 / mc(2)155) OX=246196 GN=rplE PE=1 SV=1 | **Shared** |
| A0QWT3 | metK MSMEG_3055 MSMEI_2979 | 481 | 12 | 4 | 428 | 13 | 4 | S-adenosylmethionine synthase OS=Mycolicibacterium smegmatis (strain ATCC 700084 / mc(2)155) OX=246196 GN=metK PE=1 SV=1 | **Shared** |
| A0QUZ2 | mutT1 MSMEG_2390 MSMEI_2330 | 473 | 13 | 3 | 666 | 16 | 3 | 8-oxo-(d)GTP phosphatase OS=Mycolicibacterium smegmatis (strain ATCC 700084 / mc(2)155) OX=246196 GN=mutT1 PE=1 SV=1 | **Shared** |
| A0R087 | panB MSMEG_4298 | 464 | 18 | 4 | 371 | 12 | 4 | 3-methyl-2-oxobutanoate hydroxymethyltransferase OS=Mycolicibacterium smegmatis (strain ATCC 700084 / mc(2)155) OX=246196 GN=panB PE=3 SV=1 | **Shared** |
| A0R3B8 | eno MSMEG_5415 MSMEI_5267 | 462 | 14 | 3 | 835 | 27 | 4 | Enolase OS=Mycolicibacterium smegmatis (strain ATCC 700084 / mc(2)155) OX=246196 GN=eno PE=1 SV=1 | **Shared** |
| A0QQX6 | aceA MSMEG_0911 | 447 | 19 | 4 | 289 | 14 | 4 | isocitrate lyase OS=Mycolicibacterium smegmatis (strain ATCC 700084 / mc(2)155) OX=246196 GN=aceA PE=4 SV=1 | **Shared** |
| A0R0B3 | acpM MSMEG_4326 MSMEI_4226 | 447 | 9 | 4 | 318 | 9 | 3 | Meromycolate extension acyl carrier protein OS=Mycolicibacterium smegmatis (strain ATCC 700084 / mc(2)155) OX=246196 GN=acpM PE=1 SV=1 | **Shared** |
| A0QSG6 | rpsE MSMEG_1472 MSMEI_1436 | 436 | 14 | 4 | 614 | 13 | 4 | Small ribosomal subunit protein uS5 OS=Mycolicibacterium smegmatis (strain ATCC 700084 / mc(2)155) OX=246196 GN=rpsE PE=1 SV=1 | **Shared** |
| P00761 | nrdE1 MSMEG_1019 MSMEI_0990 | 436 | 8 | 4 | 377 | 8 | 4 | Trypsin_EMS0006_Sigma OS=Sus Scrofa OX=0 GN=a PE=0 SV=0 | **Shared** |
| A0QSD8 | rplP MSMEG_1443 MSMEI_1407 | 420 | 11 | 4 | 902 | 9 | 4 | Large ribosomal subunit protein uL16 OS=Mycolicibacterium smegmatis (strain ATCC 700084 / mc(2)155) OX=246196 GN=rplP PE=1 SV=1 | **Shared** |
| A0R618 | fadD32 MSMEG_6393 MSMEI_6225 | 415 | 16 | 4 | 355 | 13 | 4 | Long-chain-fatty-acid--AMP ligase FadD32 OS=Mycolicibacterium smegmatis (strain ATCC 700084 / mc(2)155) OX=246196 GN=fadD32 PE=1 SV=1 | **Shared** |
| A0R3M4 | sucC MSMEG_5525 MSMEI_5373 | 401 | 12 | 4 | 198 | 10 | 4 | Succinate--CoA ligase [ADP-forming] subunit beta OS=Mycolicibacterium smegmatis (strain ATCC 700084 / mc(2)155) OX=246196 GN=sucC PE=1 SV=1 | **Shared** |
| A0R6Q9 | metE MSMEG_6638 | 400 | 12 | 4 | 33 | 1 | 1 | 5-methyltetrahydropteroyltriglutamate--homocysteine methyltransferase OS=Mycolicibacterium smegmatis (strain ATCC 700084 / mc(2)155) OX=246196 GN=metE PE=3 SV=1 | **Shared** |
| A0QSU3 | guaB MSMEG_1602 | 390 | 13 | 4 | 253 | 8 | 4 | Inosine-5~-monophosphate dehydrogenase OS=Mycolicibacterium smegmatis (strain ATCC 700084 / mc(2)155) OX=246196 GN=guaB PE=3 SV=1 | **Shared** |
| A0R0Y9 | lepA MSMEG_4556 | 380 | 11 | 3 | 431 | 16 | 3 | Elongation factor 4 OS=Mycolicibacterium smegmatis (strain ATCC 700084 / mc(2)155) OX=246196 GN=lepA PE=3 SV=1 | **Shared** |
| A0R730 | MSMEG_6761 | 354 | 13 | 3 | 94 | 4 | 3 | Glycerol-3-phosphate dehydrogenase OS=Mycolicibacterium smegmatis (strain ATCC 700084 / mc(2)155) OX=246196 GN=MSMEG_6761 PE=3 SV=1 | **Shared** |
| A0QS96 | rpsL MSMEG_1398 MSMEI_1360 | 342 | 18 | 4 | 462 | 15 | 4 | Small ribosomal subunit protein uS12 OS=Mycolicibacterium smegmatis (strain ATCC 700084 / mc(2)155) OX=246196 GN=rpsL PE=1 SV=1 | **Shared** |
| A0R441 | MSMEG_5696 | 339 | 16 | 4 | 204 | 9 | 4 | ~Cold-shock~ DNA-binding domain protein OS=Mycolicibacterium smegmatis (strain ATCC 700084 / mc(2)155) OX=246196 GN=MSMEG_5696 PE=4 SV=1 | **Shared** |
| A0QWW2 | gapA gap MSMEG_3084 MSMEI_3006 | 334 | 20 | 4 | 280 | 12 | 3 | Glyceraldehyde-3-phosphate dehydrogenase OS=Mycolicibacterium smegmatis (strain ATCC 700084 / mc(2)155) OX=246196 GN=gapA PE=1 SV=1 | **Shared** |
| A4ZHR8 | ahcY MSMEG_1843 MSMEI_1801 | 331 | 15 | 4 | 383 | 16 | 3 | Adenosylhomocysteinase OS=Mycolicibacterium smegmatis (strain ATCC 700084 / mc(2)155) OX=246196 GN=ahcY PE=1 SV=1 | **Shared** |
| A0R052 | MSMEG_4263 | 325 | 7 | 3 | 138 | 3 | 2 | Cytochrome bc1 complex cytochrome b subunit OS=Mycolicibacterium smegmatis (strain ATCC 700084 / mc(2)155) OX=246196 GN=MSMEG_4263 PE=1 SV=1 | **Shared** |
| A0QWY0 | tkt MSMEG_3103 | 324 | 14 | 4 | 220 | 8 | 3 | Transketolase OS=Mycolicibacterium smegmatis (strain ATCC 700084 / mc(2)155) OX=246196 GN=tkt PE=3 SV=1 | **Shared** |
| P48354 | rpoB MSMEG_1367 MSMEI_1328 | 310 | 18 | 3 | 156 | 9 | 3 | DNA gyrase subunit A OS=Mycolicibacterium smegmatis (strain ATCC 700084 / mc(2)155) OX=246196 GN=gyrA PE=1 SV=1 | **Shared** |
| A0R1V9 | MSMEG_4891 MSMEI_4766 | 307 | 12 | 4 | 326 | 11 | 4 | Alkyl hydroperoxide reductase C OS=Mycolicibacterium smegmatis (strain ATCC 700084 / mc(2)155) OX=246196 GN=MSMEG_4891 PE=1 SV=1 | **Shared** |
| A0QQV4 | MSMEG_0889 | 276 | 7 | 2 | 181 | 6 | 2 | Aldehyde dehydrogenase OS=Mycolicibacterium smegmatis (strain ATCC 700084 / mc(2)155) OX=246196 GN=MSMEG_0889 PE=3 SV=1 | **Shared** |
| A0QWS8 | mIHF msihf MSMEG_3050 MSMEI_2975 | 272 | 11 | 4 | 271 | 8 | 4 | Integration host factor OS=Mycolicibacterium smegmatis (strain ATCC 700084 / mc(2)155) OX=246196 GN=mIHF PE=1 SV=1 | **Shared** |
| A0QSD6 | rplV MSMEG_1441 MSMEI_1405 | 270 | 8 | 4 | 331 | 11 | 4 | Large ribosomal subunit protein uL22 OS=Mycolicibacterium smegmatis (strain ATCC 700084 / mc(2)155) OX=246196 GN=rplV PE=1 SV=1 | **Shared** |
| A0QSL9 | rplQ MSMEG_1525 MSMEI_1489 | 269 | 7 | 4 | 91 | 3 | 2 | Large ribosomal subunit protein bL17 OS=Mycolicibacterium smegmatis (strain ATCC 700084 / mc(2)155) OX=246196 GN=rplQ PE=1 SV=1 | **Shared** |
| A0R0B4 | MSMEG_4327 | 267 | 8 | 3 | 174 | 3 | 2 | 3-oxoacyl-[acyl-carrier-protein] synthase 1 OS=Mycolicibacterium smegmatis (strain ATCC 700084 / mc(2)155) OX=246196 GN=MSMEG_4327 PE=3 SV=1 | **Shared** |
| A0QUX8 | ilvC MSMEG_2374 MSMEI_2314 | 262 | 8 | 3 | 481 | 10 | 3 | Ketol-acid reductoisomerase (NADP(+)) OS=Mycolicibacterium smegmatis (strain ATCC 700084 / mc(2)155) OX=246196 GN=ilvC PE=1 SV=1 | **Shared** |
| A0QT08 | sdhA MSMEG_1670 | 251 | 11 | 4 | 43 | 3 | 2 | Succinate dehydrogenase flavoprotein subunit OS=Mycolicibacterium smegmatis (strain ATCC 700084 / mc(2)155) OX=246196 GN=sdhA PE=1 SV=1 | **Shared** |
| A0R576 | MSMEG_6092 | 250 | 6 | 3 | 204 | 4 | 3 | Lsr2 protein OS=Mycolicibacterium smegmatis (strain ATCC 700084 / mc(2)155) OX=246196 GN=MSMEG_6092 PE=4 SV=1 | **Shared** |
| A0R3D9 | ksgA rsmA MSMEG_5438 | 248 | 8 | 4 | 376 | 10 | 3 | Ribosomal RNA small subunit methyltransferase A OS=Mycolicibacterium smegmatis (strain ATCC 700084 / mc(2)155) OX=246196 GN=ksgA PE=3 SV=1 | **Shared** |
| A0QSS3 | groES groS MSMEG_1582 MSMEI_1544 | 246 | 10 | 4 | 122 | 4 | 3 | Co-chaperonin GroES OS=Mycolicibacterium smegmatis (strain ATCC 700084 / mc(2)155) OX=246196 GN=groES PE=1 SV=1 | **Shared** |
| A0QQ65 | MSMEG_0643 | 245 | 12 | 4 | 135 | 5 | 3 | Extracellular solute-binding protein family protein 5 putative OS=Mycolicibacterium smegmatis (strain ATCC 700084 / mc(2)155) OX=246196 GN=MSMEG_0643 PE=4 SV=1 | **Shared** |
| A0QQC5 | MSMEG_0706 | 244 | 5 | 2 | 117 | 4 | 3 | Putative 4-hydroxy-4-methyl-2-oxoglutarate aldolase OS=Mycolicibacterium smegmatis (strain ATCC 700084 / mc(2)155) OX=246196 GN=MSMEG_0706 PE=4 SV=1 | **Shared** |
| A0QYW4 | uvrA MSMEG_3808 | 242 | 9 | 2 | 596 | 18 | 3 | UvrABC system protein A OS=Mycolicibacterium smegmatis (strain ATCC 700084 / mc(2)155) OX=246196 GN=uvrA PE=3 SV=1 | **Shared** |
| A0QS62 | rplJ MSMEG_1364 MSMEI_1325 | 238 | 12 | 4 | 132 | 4 | 3 | Large ribosomal subunit protein uL10 OS=Mycolicibacterium smegmatis (strain ATCC 700084 / mc(2)155) OX=246196 GN=rplJ PE=1 SV=1 | **Shared** |
| A0QSZ3 | MSMEG_1654 | 227 | 6 | 4 | 122 | 4 | 3 | Isocitrate dehydrogenase [NADP] OS=Mycolicibacterium smegmatis (strain ATCC 700084 / mc(2)155) OX=246196 GN=MSMEG_1654 PE=1 SV=1 | **Shared** |
| A0QVY4 | MSMEG_2739 | 225 | 9 | 2 | 142 | 5 | 2 | LGFP repeat protein OS=Mycolicibacterium smegmatis (strain ATCC 700084 / mc(2)155) OX=246196 GN=MSMEG_2739 PE=4 SV=1 | **Shared** |
| A0QUV7 | etfA MSMEG_2352 | 217 | 7 | 3 | 204 | 9 | 3 | Electron transfer flavoprotein alpha subunit OS=Mycolicibacterium smegmatis (strain ATCC 700084 / mc(2)155) OX=246196 GN=etfA PE=3 SV=1 | **Shared** |
| A0QVQ3 | rpsO MSMEG_2654 MSMEI_2591 | 215 | 8 | 4 | 335 | 8 | 2 | Small ribosomal subunit protein uS15 OS=Mycolicibacterium smegmatis (strain ATCC 700084 / mc(2)155) OX=246196 GN=rpsO PE=1 SV=1 | **Shared** |
| A0R200 | atpD MSMEG_4936 MSMEI_4809 | 215 | 6 | 4 | 203 | 5 | 2 | ATP synthase subunit beta OS=Mycolicibacterium smegmatis (strain ATCC 700084 / mc(2)155) OX=246196 GN=atpD PE=1 SV=1 | **Shared** |
| A0QYU7 | rpmI MSMEG_3792 MSMEI_3704 | 213 | 4 | 4 | 140 | 3 | 3 | Large ribosomal subunit protein bL35 OS=Mycolicibacterium smegmatis (strain ATCC 700084 / mc(2)155) OX=246196 GN=rpmI PE=1 SV=1 | **Shared** |
| A0R3M3 | sucD MSMEG_5524 | 209 | 11 | 4 | 161 | 7 | 4 | Succinate--CoA ligase [ADP-forming] subunit alpha OS=Mycolicibacterium smegmatis (strain ATCC 700084 / mc(2)155) OX=246196 GN=sucD PE=3 SV=1 | **Shared** |
| A0QUV6 | etfB MSMEG_2351 | 208 | 8 | 4 | 115 | 2 | 2 | Electron transfer flavoprotein subunit beta OS=Mycolicibacterium smegmatis (strain ATCC 700084 / mc(2)155) OX=246196 GN=etfB PE=3 SV=1 | **Shared** |
| A0QSR5 | MSMEG_1574 | 200 | 7 | 3 | 84 | 2 | 2 | Glutamate decarboxylase OS=Mycolicibacterium smegmatis (strain ATCC 700084 / mc(2)155) OX=246196 GN=MSMEG_1574 PE=3 SV=1 | **Shared** |
| A0QSG3 | rpsH MSMEG_1469 MSMEI_1433 | 194 | 5 | 3 | 247 | 6 | 2 | Small ribosomal subunit protein uS8 OS=Mycolicibacterium smegmatis (strain ATCC 700084 / mc(2)155) OX=246196 GN=rpsH PE=1 SV=1 | **Shared** |
| A0R079 | glnA glnA1 MSMEG_4290 MSMEI_4189 | 194 | 11 | 4 | 457 | 13 | 2 | Glutamine synthetase OS=Mycolicibacterium smegmatis (strain ATCC 700084 / mc(2)155) OX=246196 GN=glnA PE=1 SV=1 | **Shared** |
| A0QVQ5 | pnp gpsI MSMEG_2656 MSMEI_2593 | 188 | 9 | 2 | 22 | 1 | 1 | Polyribonucleotide nucleotidyltransferase OS=Mycolicibacterium smegmatis (strain ATCC 700084 / mc(2)155) OX=246196 GN=pnp PE=1 SV=1 | **Shared** |
| A0QSL3 | infA MSMEG_1519 MSMEI_1483 | 182 | 9 | 4 | 194 | 7 | 3 | Translation initiation factor IF-1 OS=Mycolicibacterium smegmatis (strain ATCC 700084 / mc(2)155) OX=246196 GN=infA PE=1 SV=1 | **Shared** |
| A0R7F9 | rpsF MSMEG_6897 MSMEI_6713 | 180 | 6 | 3 | 229 | 6 | 3 | Small ribosomal subunit protein bS6 OS=Mycolicibacterium smegmatis (strain ATCC 700084 / mc(2)155) OX=246196 GN=rpsF PE=1 SV=1 | **Shared** |
| A0R2T1 | MSMEG_5223 | 179 | 7 | 4 | 341 | 10 | 3 | DUF6542 domain-containing protein OS=Mycolicibacterium smegmatis (strain ATCC 700084 / mc(2)155) OX=246196 GN=MSMEG_5223 PE=4 SV=1 | **Shared** |
| A0QR89 | MSMEG_1028 MSMEG_2308 | 178 | 6 | 3 | 243 | 9 | 2 | Geranylgeranyl reductase OS=Mycolicibacterium smegmatis (strain ATCC 700084 / mc(2)155) OX=246196 GN=MSMEG_1028 PE=4 SV=1 | **Shared** |
| A0R051 | MSMEG_4262 | 178 | 7 | 3 | 179 | 7 | 3 | Cytochrome bc1 complex Rieske iron-sulfur subunit OS=Mycolicibacterium smegmatis (strain ATCC 700084 / mc(2)155) OX=246196 GN=MSMEG_4262 PE=1 SV=1 | **Shared** |
| A0QTK6 | hpf MSMEG_1878 | 177 | 7 | 3 | 660 | 13 | 2 | Ribosome hibernation promoting factor OS=Mycolicibacterium smegmatis (strain ATCC 700084 / mc(2)155) OX=246196 GN=hpf PE=1 SV=1 | **Shared** |
| A0QQH1 | MSMEG_0753 | 173 | 5 | 2 | 193 | 4 | 3 | DUF4878 domain-containing protein OS=Mycolicibacterium smegmatis (strain ATCC 700084 / mc(2)155) OX=246196 GN=MSMEG_0753 PE=4 SV=1 | **Shared** |
| A0R0S4 | uppS MSMEG_4490 MSMEI_4379 | 173 | 6 | 2 | 270 | 8 | 3 | Decaprenyl diphosphate synthase OS=Mycolicibacterium smegmatis (strain ATCC 700084 / mc(2)155) OX=246196 GN=uppS PE=1 SV=1 | **Shared** |
| A0R5H1 | MSMEG_6189 | 161 | 6 | 2 | 99 | 5 | 3 | Transcriptional regulator Crp/Fnr family protein OS=Mycolicibacterium smegmatis (strain ATCC 700084 / mc(2)155) OX=246196 GN=MSMEG_6189 PE=4 SV=1 | **Shared** |
| A0R461 | MSMEG_5715 | 158 | 4 | 3 | 163 | 4 | 4 | Luciferase-like domain-containing protein OS=Mycolicibacterium smegmatis (strain ATCC 700084 / mc(2)155) OX=246196 GN=MSMEG_5715 PE=4 SV=1 | **Shared** |
| A0QUX1 | gatB MSMEG_2367 MSMEI_2307 | 156 | 7 | 3 | 237 | 7 | 2 | Aspartyl/glutamyl-tRNA(Asn/Gln) amidotransferase subunit B OS=Mycolicibacterium smegmatis (strain ATCC 700084 / mc(2)155) OX=246196 GN=gatB PE=3 SV=1 | **Shared** |
| A0R198 | clpP MSMEG_4673 | 153 | 3 | 3 | 221 | 5 | 4 | ATP-dependent Clp protease proteolytic subunit OS=Mycolicibacterium smegmatis (strain ATCC 700084 / mc(2)155) OX=246196 GN=clpP PE=1 SV=1 | **Shared** |
| A0QX20 | acnA acn MSMEG_3143 MSMEI_3062 | 151 | 10 | 4 | 196 | 9 | 4 | Aconitate hydratase A OS=Mycolicibacterium smegmatis (strain ATCC 700084 / mc(2)155) OX=246196 GN=acnA PE=1 SV=1 | **Shared** |
| A0QS41 | MSMEG_1341 | 150 | 4 | 4 | 55 | 2 | 2 | MaoC family protein OS=Mycolicibacterium smegmatis (strain ATCC 700084 / mc(2)155) OX=246196 GN=MSMEG_1341 PE=1 SV=1 | **Shared** |
| A0R008 | sepF MSMEG_4219 MSMEI_4121 | 150 | 4 | 2 | 42 | 2 | 1 | Cell division protein SepF OS=Mycolicibacterium smegmatis (strain ATCC 700084 / mc(2)155) OX=246196 GN=sepF PE=3 SV=1 | **Shared** |
| A0R072 | sucB MSMEG_4283 | 147 | 4 | 3 | 186 | 7 | 4 | Dihydrolipoamide acetyltransferase component of pyruvate dehydrogenase complex OS=Mycolicibacterium smegmatis (strain ATCC 700084 / mc(2)155) OX=246196 GN=sucB PE=3 SV=1 | **Shared** |
| A0R1H2 | MSMEG_4752 | 142 | 3 | 3 | 34 | 1 | 1 | DUF3618 domain-containing protein OS=Mycolicibacterium smegmatis (strain ATCC 700084 / mc(2)155) OX=246196 GN=MSMEG_4752 PE=4 SV=1 | **Shared** |
| A0QSZ1 | MSMEG_1652 | 140 | 4 | 4 | 145 | 3 | 3 | O-acetylhomoserine sulfhydrylase OS=Mycolicibacterium smegmatis (strain ATCC 700084 / mc(2)155) OX=246196 GN=MSMEG_1652 PE=3 SV=1 | **Shared** |
| A0R4C9 | MSMEG_5789 MSMEI_5636 | 140 | 5 | 3 | 70 | 2 | 2 | Putative thiosulfate sulfurtransferase OS=Mycolicibacterium smegmatis (strain ATCC 700084 / mc(2)155) OX=246196 GN=MSMEG_5789 PE=1 SV=1 | **Shared** |
| A0R061 | MSMEG_4272 | 136 | 2 | 2 | 202 | 4 | 3 | HesB/YadR/YfhF family protein OS=Mycolicibacterium smegmatis (strain ATCC 700084 / mc(2)155) OX=246196 GN=MSMEG_4272 PE=4 SV=1 | **Shared** |
| A0QV03 | rpmB MSMEG_2400 | 133 | 4 | 4 | 184 | 5 | 4 | Large ribosomal subunit protein bL28 OS=Mycolicibacterium smegmatis (strain ATCC 700084 / mc(2)155) OX=246196 GN=rpmB PE=1 SV=1 | **Shared** |
| A0R561 | carD MSMEG_6077 MSMEI_5917 | 133 | 2 | 2 | 82 | 2 | 2 | RNA polymerase-binding transcription factor CarD OS=Mycolicibacterium smegmatis (strain ATCC 700084 / mc(2)155) OX=246196 GN=carD PE=1 SV=1 | **Shared** |
| A0R417 | gltA MSMEG_5672 | 131 | 5 | 2 | 169 | 6 | 3 | Citrate synthase OS=Mycolicibacterium smegmatis (strain ATCC 700084 / mc(2)155) OX=246196 GN=gltA PE=3 SV=1 | **Shared** |
| A0QVB9 | tsf MSMEG_2520 MSMEI_2461 | 120 | 6 | 2 | 53 | 3 | 1 | Elongation factor Ts OS=Mycolicibacterium smegmatis (strain ATCC 700084 / mc(2)155) OX=246196 GN=tsf PE=1 SV=1 | **Shared** |
| A0R623 | MSMEG_6398 | 114 | 2 | 2 | 23 | 1 | 1 | Antigen 85-A OS=Mycolicibacterium smegmatis (strain ATCC 700084 / mc(2)155) OX=246196 GN=MSMEG_6398 PE=4 SV=1 | **Shared** |
| A0R202 | atpA MSMEG_4938 MSMEI_4811 | 113 | 9 | 3 | 80 | 4 | 3 | ATP synthase subunit alpha OS=Mycolicibacterium smegmatis (strain ATCC 700084 / mc(2)155) OX=246196 GN=atpA PE=1 SV=1 | **Shared** |
| A0QT20 | MSMEG_1682 | 112 | 4 | 3 | 91 | 2 | 2 | Flavin-containing monooxygenase FMO OS=Mycolicibacterium smegmatis (strain ATCC 700084 / mc(2)155) OX=246196 GN=MSMEG_1682 PE=4 SV=1 | **Shared** |
| A0R2Y1 | MSMEG_5273 | 104 | 5 | 3 | 137 | 5 | 3 | Beta-ketoadipyl CoA thiolase OS=Mycolicibacterium smegmatis (strain ATCC 700084 / mc(2)155) OX=246196 GN=MSMEG_5273 PE=3 SV=1 | **Shared** |
| A0R3D2 | rplY ctc MSMEG_5431 | 104 | 4 | 2 | 38 | 2 | 1 | Large ribosomal subunit protein bL25 OS=Mycolicibacterium smegmatis (strain ATCC 700084 / mc(2)155) OX=246196 GN=rplY PE=1 SV=1 | **Shared** |
| A0R557 | MSMEG_6073 MSMEI_5913 | 101 | 2 | 1 | 166 | 5 | 2 | Uncharacterized tRNA/rRNA methyltransferase MSMEG_6073/MSMEI_5913 OS=Mycolicibacterium smegmatis (strain ATCC 700084 / mc(2)155) OX=246196 GN=MSMEG_6073 PE=3 SV=1 | **Shared** |
| A0QP17 | MSMEG_0240 | 96 | 4 | 3 | 140 | 3 | 2 | Transcriptional regulator OS=Mycolicibacterium smegmatis (strain ATCC 700084 / mc(2)155) OX=246196 GN=MSMEG_0240 PE=4 SV=1 | **Shared** |
| A0QUY2 | serA MSMEG_2378 | 91 | 4 | 2 | 15 | 1 | 1 | D-3-phosphoglycerate dehydrogenase OS=Mycolicibacterium smegmatis (strain ATCC 700084 / mc(2)155) OX=246196 GN=serA PE=3 SV=1 | **Shared** |
| A0R199 | tig MSMEG_4674 MSMEI_4557 | 86 | 3 | 3 | 173 | 4 | 3 | Trigger factor OS=Mycolicibacterium smegmatis (strain ATCC 700084 / mc(2)155) OX=246196 GN=tig PE=1 SV=1 | **Shared** |
| A0R7J6 | MSMEG_6941 | 86 | 4 | 3 | 63 | 3 | 2 | R3H domain-containing protein OS=Mycolicibacterium smegmatis (strain ATCC 700084 / mc(2)155) OX=246196 GN=MSMEG_6941 PE=4 SV=1 | **Shared** |
| A0QQF0 | clpB MSMEG_0732 | 84 | 3 | 3 | 58 | 2 | 2 | Chaperone protein ClpB OS=Mycolicibacterium smegmatis (strain ATCC 700084 / mc(2)155) OX=246196 GN=clpB PE=3 SV=1 | **Shared** |
| A0R201 | atpG MSMEG_4937 MSMEI_4810 | 84 | 3 | 3 | 23 | 1 | 1 | ATP synthase gamma chain OS=Mycolicibacterium smegmatis (strain ATCC 700084 / mc(2)155) OX=246196 GN=atpG PE=1 SV=1 | **Shared** |
| A0QVL2 | mqo MSMEG_2613 MSMEI_2551 | 83 | 3 | 2 | 25 | 1 | 1 | Probable malate:quinone oxidoreductase OS=Mycolicibacterium smegmatis (strain ATCC 700084 / mc(2)155) OX=246196 GN=mqo PE=1 SV=1 | **Shared** |
| A0R3D6 | MSMEG_5435 MSMEI_5285 | 81 | 6 | 3 | 172 | 5 | 4 | Putative ligase MSMEG_5435/MSMEI_5285 OS=Mycolicibacterium smegmatis (strain ATCC 700084 / mc(2)155) OX=246196 GN=MSMEG_5435 PE=1 SV=1 | **Shared** |
| A0QWW3 | pgk MSMEG_3085 MSMEI_3007 | 80 | 3 | 1 | 28 | 1 | 1 | Phosphoglycerate kinase OS=Mycolicibacterium smegmatis (strain ATCC 700084 / mc(2)155) OX=246196 GN=pgk PE=1 SV=1 | **Shared** |
| A0R465 | MSMEG_5720 | 79 | 4 | 2 | 34 | 1 | 1 | 3-hydroxyacyl-CoA dehydrogenase OS=Mycolicibacterium smegmatis (strain ATCC 700084 / mc(2)155) OX=246196 GN=MSMEG_5720 PE=3 SV=1 | **Shared** |
| A0R610 | dprE2 MSMEG_6385 MSMEI_6217 LJ00_31560 | 79 | 3 | 2 | 75 | 2 | 2 | Decaprenylphosphoryl-2-keto-beta-D-erythro-pentose reductase OS=Mycolicibacterium smegmatis (strain ATCC 700084 / mc(2)155) OX=246196 GN=dprE2 PE=1 SV=1 | **Shared** |
| A0R2B1 | kgd sucA MSMEG_5049 MSMEI_4922 | 78 | 3 | 2 | 137 | 7 | 2 | Multifunctional 2-oxoglutarate metabolism enzyme OS=Mycolicibacterium smegmatis (strain ATCC 700084 / mc(2)155) OX=246196 GN=kgd PE=1 SV=1 | **Shared** |
| A0QSB1 | MSMEG_1416 | 77 | 2 | 2 | 44 | 1 | 1 | Pyridine nucleotide-disulphide oxidoreductase OS=Mycolicibacterium smegmatis (strain ATCC 700084 / mc(2)155) OX=246196 GN=MSMEG_1416 PE=4 SV=1 | **Shared** |
| A0R2J2 | MSMEG_5134 | 77 | 2 | 2 | 25 | 1 | 1 | Uncharacterized protein OS=Mycolicibacterium smegmatis (strain ATCC 700084 / mc(2)155) OX=246196 GN=MSMEG_5134 PE=4 SV=1 | **Shared** |
| A0QSD9 | rpmC MSMEG_1444 MSMEI_1408 | 76 | 3 | 3 | 186 | 4 | 3 | Large ribosomal subunit protein uL29 OS=Mycolicibacterium smegmatis (strain ATCC 700084 / mc(2)155) OX=246196 GN=rpmC PE=1 SV=1 | **Shared** |
| A0QX24 | moxR MSMEG_3147 | 76 | 3 | 3 | 45 | 2 | 2 | ATPase MoxR family protein OS=Mycolicibacterium smegmatis (strain ATCC 700084 / mc(2)155) OX=246196 GN=moxR PE=4 SV=1 | **Shared** |
| A0R197 | clpP MSMEG_4672 | 71 | 3 | 3 | 27 | 1 | 1 | ATP-dependent Clp protease proteolytic subunit OS=Mycolicibacterium smegmatis (strain ATCC 700084 / mc(2)155) OX=246196 GN=clpP PE=3 SV=1 | **Shared** |
| A0QYW6 | MSMEG_3811 | 70 | 2 | 2 | 122 | 5 | 3 | Universal stress protein family protein putative OS=Mycolicibacterium smegmatis (strain ATCC 700084 / mc(2)155) OX=246196 GN=MSMEG_3811 PE=1 SV=1 | **Shared** |
| A0QYZ2 | polA MSMEG_3839 | 70 | 2 | 1 | 20 | 2 | 1 | DNA polymerase I OS=Mycolicibacterium smegmatis (strain ATCC 700084 / mc(2)155) OX=246196 GN=polA PE=1 SV=1 | **Shared** |
| A0QQX8 | MSMEG_0913 | 69 | 4 | 3 | 53 | 2 | 1 | Methoxy mycolic acid synthase 1 OS=Mycolicibacterium smegmatis (strain ATCC 700084 / mc(2)155) OX=246196 GN=MSMEG_0913 PE=3 SV=1 | **Shared** |
| A0QS64 | MSMEG_1366 | 68 | 2 | 2 | 28 | 1 | 1 | ABC transporter ATP-binding protein OS=Mycolicibacterium smegmatis (strain ATCC 700084 / mc(2)155) OX=246196 GN=MSMEG_1366 PE=1 SV=1 | **Shared** |
| A0QYS6 | argG MSMEG_3770 MSMEI_3681 | 67 | 3 | 2 | 110 | 2 | 2 | Argininosuccinate synthase OS=Mycolicibacterium smegmatis (strain ATCC 700084 / mc(2)155) OX=246196 GN=argG PE=3 SV=1 | **Shared** |
| P71534 | MSMEG_0919 | 67 | 2 | 2 | 42 | 1 | 1 | 3-oxoacyl-[acyl-carrier-protein] reductase MabA OS=Mycolicibacterium smegmatis (strain ATCC 700084 / mc(2)155) OX=246196 GN=mabA PE=1 SV=2 | **Shared** |
| A0R616 | MSMEG_6391 | 65 | 4 | 1 | 77 | 2 | 2 | Propionyl-CoA carboxylase beta chain OS=Mycolicibacterium smegmatis (strain ATCC 700084 / mc(2)155) OX=246196 GN=MSMEG_6391 PE=3 SV=1 | **Shared** |
| A0QU54 | MSMEG_2081 | 63 | 3 | 1 | 40 | 2 | 1 | Acyl-CoA dehydrogenase OS=Mycolicibacterium smegmatis (strain ATCC 700084 / mc(2)155) OX=246196 GN=MSMEG_2081 PE=3 SV=1 | **Shared** |
| A0R0R9 | glyS glyQS MSMEG_4485 | 63 | 3 | 2 | 127 | 5 | 3 | Glycine--tRNA ligase OS=Mycolicibacterium smegmatis (strain ATCC 700084 / mc(2)155) OX=246196 GN=glyS PE=3 SV=1 | **Shared** |
| A0QQC8 | dnaK MSMEG_0709 MSMEI_0692 | 61 | 3 | 2 | 68 | 2 | 2 | Chaperone protein DnaK OS=Mycolicibacterium smegmatis (strain ATCC 700084 / mc(2)155) OX=246196 GN=dnaK PE=1 SV=1 | **Shared** |
| A0QRS9 | MSMEG_1225 | 60 | 2 | 1 | 40 | 2 | 1 | DUF2786 domain-containing protein OS=Mycolicibacterium smegmatis (strain ATCC 700084 / mc(2)155) OX=246196 GN=MSMEG_1225 PE=4 SV=1 | **Shared** |
| A0QQW5 | MSMEG_0900 | 57 | 2 | 2 | 22 | 1 | 1 | Eptc-inducible aldehyde dehydrogenase OS=Mycolicibacterium smegmatis (strain ATCC 700084 / mc(2)155) OX=246196 GN=MSMEG_0900 PE=3 SV=1 | **Shared** |
| A0QPG3 | tylF MSMEG_0388 | 56 | 2 | 2 | 19 | 1 | 1 | Macrocin-O-methyltransferase OS=Mycolicibacterium smegmatis (strain ATCC 700084 / mc(2)155) OX=246196 GN=tylF PE=4 SV=1 | **Shared** |
| P0CG99 | KRT10 KPP | 56 | 2 | 1 | 66 | 2 | 2 | Ribonucleoside-diphosphate reductase subunit alpha 1 OS=Mycolicibacterium smegmatis (strain ATCC 700084 / mc(2)155) OX=246196 GN=nrdE1 PE=1 SV=1 | **Shared** |
| A0R0I3 | MSMEG_4396 | 55 | 3 | 3 | 26 | 1 | 1 | Isochorismatase hydrolase OS=Mycolicibacterium smegmatis (strain ATCC 700084 / mc(2)155) OX=246196 GN=MSMEG_4396 PE=4 SV=1 | **Shared** |
| A0R2C0 | MSMEG_5058 | 55 | 2 | 2 | 22 | 1 | 1 | ABC transporter ATP-binding protein SugC OS=Mycolicibacterium smegmatis (strain ATCC 700084 / mc(2)155) OX=246196 GN=MSMEG_5058 PE=1 SV=1 | **Shared** |
| A0R597 | ppa MSMEG_6114 | 53 | 2 | 2 | 55 | 2 | 2 | Inorganic pyrophosphatase OS=Mycolicibacterium smegmatis (strain ATCC 700084 / mc(2)155) OX=246196 GN=ppa PE=3 SV=1 | **Shared** |
| A0QYQ1 | rluB MSMEG_3740 | 52 | 1 | 1 | 86 | 2 | 1 | Pseudouridine synthase OS=Mycolicibacterium smegmatis (strain ATCC 700084 / mc(2)155) OX=246196 GN=rluB PE=3 SV=1 | **Shared** |
| A0QTQ8 | MSMEG_1930 | 51 | 1 | 1 | 140 | 4 | 3 | RNA helicase OS=Mycolicibacterium smegmatis (strain ATCC 700084 / mc(2)155) OX=246196 GN=MSMEG_1930 PE=3 SV=1 | **Shared** |
| A0R4D7 | MSMEG_5797 | 51 | 2 | 2 | 17 | 1 | 1 | DUF3073 domain-containing protein OS=Mycolicibacterium smegmatis (strain ATCC 700084 / mc(2)155) OX=246196 GN=MSMEG_5797 PE=4 SV=1 | **Shared** |
| A0QPG6 | MSMEG_0391 | 46 | 1 | 1 | 75 | 2 | 2 | Rmt3 protein OS=Mycolicibacterium smegmatis (strain ATCC 700084 / mc(2)155) OX=246196 GN=MSMEG_0391 PE=4 SV=1 | **Shared** |
| A0QS45 | rplK MSMEG_1346 MSMEI_1308 | 46 | 2 | 2 | 58 | 4 | 1 | Large ribosomal subunit protein uL11 OS=Mycolicibacterium smegmatis (strain ATCC 700084 / mc(2)155) OX=246196 GN=rplK PE=1 SV=1 | **Shared** |
| A0QVM3 | rimP MSMEG_2624 MSMEI_2562 | 46 | 2 | 1 | 34 | 1 | 1 | Ribosome maturation factor RimP OS=Mycolicibacterium smegmatis (strain ATCC 700084 / mc(2)155) OX=246196 GN=rimP PE=1 SV=1 | **Shared** |
| A0QS63 | rplL MSMEG_1365 | 44 | 2 | 2 | 89 | 3 | 2 | Large ribosomal subunit protein bL12 OS=Mycolicibacterium smegmatis (strain ATCC 700084 / mc(2)155) OX=246196 GN=rplL PE=3 SV=1 | **Shared** |
| A0R6D7 | MSMEG_6512 | 43 | 1 | 1 | 27 | 1 | 1 | Acyl-CoA dehydrogenase domain protein OS=Mycolicibacterium smegmatis (strain ATCC 700084 / mc(2)155) OX=246196 GN=MSMEG_6512 PE=3 SV=1 | **Shared** |
| A0QQX7 | MSMEG_0912 | 42 | 1 | 1 | 33 | 1 | 1 | 3-hydroxybutyryl-CoA dehydrogenase OS=Mycolicibacterium smegmatis (strain ATCC 700084 / mc(2)155) OX=246196 GN=MSMEG_0912 PE=4 SV=1 | **Shared** |
| A0R3N9 | pgi MSMEG_5541 MSMEI_5387 | 40 | 1 | 1 | 23 | 2 | 1 | Glucose-6-phosphate isomerase OS=Mycolicibacterium smegmatis (strain ATCC 700084 / mc(2)155) OX=246196 GN=pgi PE=1 SV=1 | **Shared** |
| A0QS49 | MSMEG_1350 | 39 | 2 | 1 | 58 | 3 | 1 | Cyclopropane-fatty-acyl-phospholipid synthase 1 OS=Mycolicibacterium smegmatis (strain ATCC 700084 / mc(2)155) OX=246196 GN=MSMEG_1350 PE=3 SV=1 | **Shared** |
| A0R2V7 | glyA MSMEG_5249 MSMEI_5111 | 39 | 1 | 1 | 29 | 1 | 1 | Serine hydroxymethyltransferase OS=Mycolicibacterium smegmatis (strain ATCC 700084 / mc(2)155) OX=246196 GN=glyA PE=1 SV=1 | **Shared** |
| A0R517 | MSMEG_6032 | 39 | 2 | 1 | 98 | 3 | 2 | Transcriptional regulator TetR family protein OS=Mycolicibacterium smegmatis (strain ATCC 700084 / mc(2)155) OX=246196 GN=MSMEG_6032 PE=4 SV=1 | **Shared** |
| A0R656 | MSMEG_6431 | 38 | 1 | 1 | 34 | 1 | 1 | HTH cro/C1-type domain-containing protein OS=Mycolicibacterium smegmatis (strain ATCC 700084 / mc(2)155) OX=246196 GN=MSMEG_6431 PE=4 SV=1 | **Shared** |
| A0QNF6 | MSMEG_0024 | 37 | 1 | 1 | 34 | 2 | 1 | Peptidyl-prolyl cis-trans isomerase OS=Mycolicibacterium smegmatis (strain ATCC 700084 / mc(2)155) OX=246196 GN=MSMEG_0024 PE=3 SV=1 | **Shared** |
| A0R7F6 | rplI MSMEG_6894 MSMEI_6710 | 35 | 1 | 1 | 109 | 3 | 3 | Large ribosomal subunit protein bL9 OS=Mycolicibacterium smegmatis (strain ATCC 700084 / mc(2)155) OX=246196 GN=rplI PE=1 SV=1 | **Shared** |
| A0QNE0 | gyrB MSMEG_0005 MSMEI_0007 | 34 | 1 | 1 | 107 | 3 | 2 | DNA gyrase subunit B OS=Mycolicibacterium smegmatis (strain ATCC 700084 / mc(2)155) OX=246196 GN=gyrB PE=1 SV=1 | **Shared** |
| A0QPN1 | topoM MSMEG_0456 MSMEI_0443 | 34 | 1 | 1 | 47 | 3 | 1 | Topoisomerase subunit TopoM OS=Mycolicibacterium smegmatis (strain ATCC 700084 / mc(2)155) OX=246196 GN=topoM PE=1 SV=1 | **Shared** |
| A0R588 | ftsH MSMEG_6105 | 34 | 2 | 1 | 462 | 13 | 2 | ATP-dependent zinc metalloprotease FtsH OS=Mycolicibacterium smegmatis (strain ATCC 700084 / mc(2)155) OX=246196 GN=ftsH PE=3 SV=1 | **Shared** |
| A0QXZ5 | tpx MSMEG_3479 | 33 | 1 | 1 | 41 | 1 | 1 | Thiol peroxidase OS=Mycolicibacterium smegmatis (strain ATCC 700084 / mc(2)155) OX=246196 GN=tpx PE=3 SV=1 | **Shared** |
| A0QP32 | pckG MSMEG_0255 MSMEI_0248 | 30 | 2 | 1 | 54 | 2 | 2 | Phosphoenolpyruvate carboxykinase [GTP] OS=Mycolicibacterium smegmatis (strain ATCC 700084 / mc(2)155) OX=246196 GN=pckG PE=1 SV=1 | **Shared** |
| Q9RP48 | hup cipMa hlp MSMEG_2389 MSMEI_2329 | 27 | 1 | 1 | 29 | 3 | 1 | Alpha-14-glucan:maltose-1-phosphate maltosyltransferase OS=Mycolicibacterium smegmatis (strain ATCC 700084 / mc(2)155) OX=246196 GN=glgE PE=1 SV=1 | **Shared** |
| A0R429 | serC MSMEG_5684 | 26 | 1 | 1 | 19 | 1 | 1 | Phosphoserine aminotransferase OS=Mycolicibacterium smegmatis (strain ATCC 700084 / mc(2)155) OX=246196 GN=serC PE=3 SV=1 | **Shared** |
| A0R7J7 | MSMEG_6942 | 26 | 1 | 1 | 74 | 4 | 2 | Membrane protein insertase YidC OS=Mycolicibacterium smegmatis (strain ATCC 700084 / mc(2)155) OX=246196 GN=MSMEG_6942 PE=3 SV=1 | **Shared** |
| A0QRA8 | MSMEG_1049 MSMEG_2329 | 25 | 1 | 1 | 153 | 5 | 2 | Methyltransferase UbiE/COQ5 family protein OS=Mycolicibacterium smegmatis (strain ATCC 700084 / mc(2)155) OX=246196 GN=MSMEG_1049 PE=4 SV=1 | **Shared** |
| A0QSY1 | MSMEG_1642 | 25 | 1 | 1 | 178 | 6 | 2 | ABC transporter ATP-binding protein OS=Mycolicibacterium smegmatis (strain ATCC 700084 / mc(2)155) OX=246196 GN=MSMEG_1642 PE=4 SV=1 | **Shared** |
| A0QU63 | smpB MSMEG_2091 MSMEI_2045 | 25 | 1 | 1 | 44 | 3 | 2 | SsrA-binding protein OS=Mycolicibacterium smegmatis (strain ATCC 700084 / mc(2)155) OX=246196 GN=smpB PE=1 SV=1 | **Shared** |
| A0R2B0 | MSMEG_5048 | 25 | 1 | 1 | 19 | 1 | 1 | EcsC protein family protein OS=Mycolicibacterium smegmatis (strain ATCC 700084 / mc(2)155) OX=246196 GN=MSMEG_5048 PE=4 SV=1 | **Shared** |
| A0R0B2 | fabD MSMEG_4325 MSMEI_4225 | 24 | 1 | 1 | 33 | 1 | 1 | Malonyl CoA-acyl carrier protein transacylase OS=Mycolicibacterium smegmatis (strain ATCC 700084 / mc(2)155) OX=246196 GN=fabD PE=1 SV=1 | **Shared** |
| A0QRP5 | MSMEG_1191 | 23 | 1 | 1 | 27 | 1 | 1 | DUF5652 domain-containing protein OS=Mycolicibacterium smegmatis (strain ATCC 700084 / mc(2)155) OX=246196 GN=MSMEG_1191 PE=4 SV=1 | **Shared** |
| A0R120 | MSMEG_4589 | 23 | 1 | 1 | 58 | 2 | 2 | Rhodanese domain protein/cystathionine beta-lyase OS=Mycolicibacterium smegmatis (strain ATCC 700084 / mc(2)155) OX=246196 GN=MSMEG_4589 PE=4 SV=1 | **Shared** |
| A0R5N8 | MSMEG_6257 | 23 | 1 | 1 | 34 | 2 | 2 | Aspartokinase OS=Mycolicibacterium smegmatis (strain ATCC 700084 / mc(2)155) OX=246196 GN=MSMEG_6257 PE=3 SV=1 | **Shared** |
| Q3I5Q7 | ssb MSMEG_6896 MSMEI_6712 | 23 | 1 | 1 | 73 | 3 | 2 | HBHA-like protein OS=Mycolicibacterium smegmatis (strain ATCC 700084 / mc(2)155) OX=246196 GN=MSMEG_0919 PE=4 SV=1 | **Shared** |
| A0QND6 | dnaN MSMEG_0001 MSMEI_0003 | 22 | 1 | 1 | 21 | 1 | 1 | Beta sliding clamp OS=Mycolicibacterium smegmatis (strain ATCC 700084 / mc(2)155) OX=246196 GN=dnaN PE=1 SV=1 | **Shared** |
| A0QNJ7 | MSMEG_0067 | 22 | 1 | 1 | 13 | 1 | 1 | CobQ/CobB/MinD/ParA nucleotide binding domain-containing protein OS=Mycolicibacterium smegmatis (strain ATCC 700084 / mc(2)155) OX=246196 GN=MSMEG_0067 PE=4 SV=1 | **Shared** |
| A0QU51 | fbpB MSMEG_2078 MSMEI_2033 | 22 | 1 | 1 | 21 | 1 | 1 | Diacylglycerol acyltransferase/mycolyltransferase Ag85B OS=Mycolicibacterium smegmatis (strain ATCC 700084 / mc(2)155) OX=246196 GN=fbpB PE=2 SV=1 | **Shared** |
| A0QT19 | MSMEG_1681 | 21 | 1 | 1 | 30 | 1 | 1 | Endoribonuclease L-PSP superfamily protein OS=Mycolicibacterium smegmatis (strain ATCC 700084 / mc(2)155) OX=246196 GN=MSMEG_1681 PE=4 SV=1 | **Shared** |
| A0R3C8 | prs MSMEG_5427 | 21 | 1 | 1 | 16 | 1 | 1 | Ribose-phosphate pyrophosphokinase OS=Mycolicibacterium smegmatis (strain ATCC 700084 / mc(2)155) OX=246196 GN=prs PE=1 SV=1 | **Shared** |
| A0R633 | MSMEG_6408 | 21 | 1 | 1 | 136 | 4 | 2 | Acyltransferase family protein OS=Mycolicibacterium smegmatis (strain ATCC 700084 / mc(2)155) OX=246196 GN=MSMEG_6408 PE=4 SV=1 | **Shared** |
| A0R1Y7 | MSMEG_4920 MSMEI_4793 | 20 | 1 | 1 | 73 | 3 | 2 | Probable acetyl-CoA acetyltransferase OS=Mycolicibacterium smegmatis (strain ATCC 700084 / mc(2)155) OX=246196 GN=MSMEG_4920 PE=1 SV=2 | **Shared** |
| A0QQJ4 | fgd fgd1 MSMEG_0777 MSMEI_0761 | 19 | 2 | 1 | 94 | 4 | 3 | F420-dependent glucose-6-phosphate dehydrogenase OS=Mycolicibacterium smegmatis (strain ATCC 700084 / mc(2)155) OX=246196 GN=fgd PE=1 SV=2 | **Shared** |
| A0R7K0 | rpmH MSMEG_6946 MSMEI_6752 | 19 | 1 | 1 | 53 | 2 | 2 | Large ribosomal subunit protein bL34 OS=Mycolicibacterium smegmatis (strain ATCC 700084 / mc(2)155) OX=246196 GN=rpmH PE=1 SV=1 | **Shared** |
| A0R006 | wag31 ag84 MSMEG_4217 MSMEI_4119 | 18 | 1 | 1 | 52 | 1 | 1 | Cell wall synthesis protein Wag31 OS=Mycolicibacterium smegmatis (strain ATCC 700084 / mc(2)155) OX=246196 GN=wag31 PE=1 SV=1 | **Shared** |
| A0R5K1 | MSMEG_6220 | 17 | 1 | 1 | 19 | 1 | 1 | Lipoprotein OS=Mycolicibacterium smegmatis (strain ATCC 700084 / mc(2)155) OX=246196 GN=MSMEG_6220 PE=4 SV=1 | **Shared** |
| A0QNY0 | MSMEG_0203 MSMEG_0398 MSMEG_0801 MSMEG_1002 MSMEG_1257 MSMEG_1405 MSMEG_2002 MSMEG_2282 MSMEG_2829 MSMEG_3165 MSMEG_3696 MSMEG_4402 MSMEG_4791 MSMEG_4927 MSMEG_4946 MSMEG_5093 MSMEG_5379 MSMEG_6149 MSMEG_6156 MSMEG_6162 MSMEG_6463 | 16 | 1 | 1 | 87 | 3 | 1 | IS1096 tnpR protein OS=Mycolicibacterium smegmatis (strain ATCC 700084 / mc(2)155) OX=246196 GN=MSMEG_0203 PE=4 SV=1 | **Shared** |
| A0QSF9 | rplN MSMEG_1465 MSMEI_1429 | 16 | 1 | 1 | 78 | 2 | 2 | Large ribosomal subunit protein uL14 OS=Mycolicibacterium smegmatis (strain ATCC 700084 / mc(2)155) OX=246196 GN=rplN PE=1 SV=1 | **Shared** |
| A0R221 | MSMEG_4957 | 16 | 1 | 1 | 29 | 1 | 1 | Homoserine dehydrogenase OS=Mycolicibacterium smegmatis (strain ATCC 700084 / mc(2)155) OX=246196 GN=MSMEG_4957 PE=3 SV=1 | **Shared** |
